# Identification of novel natural drug candidates against BRAF mutated carcinoma; An integrative in-silico structure-based pharmacophore modeling and virtual screening process

**DOI:** 10.3389/fchem.2022.986376

**Published:** 2022-10-04

**Authors:** F. A. Dain Md Opo, Ahad Amer Alsaiari, Mohammad Habibur Rahman Molla, Md Afsar Ahmed Sumon, Khaled A. Yaghmour, Foysal Ahammad, Farhan Mohammad, Jesus Simal-Gandara

**Affiliations:** ^1^ Department of Biological Science, Faculty of Sciences, King Abdulaziz University (KAU), Jeddah, Saudi Arabia; ^2^ Embryonic Stem Cell Research Unit, King Fahd Medical Research Center (KFMRC), KAU, Jeddah, Saudi Arabia; ^3^ Clinical Laboratories, Science Department, College of Applied Medical Science, Taif University, Taif, Saudi Arabia; ^4^ Department of Marine Biology, Faculty of Marine Sciences, King Abdulaziz University, Jeddah, Saudi Arabia; ^5^ Family Medicine Department, Faculty of Medicine, King Abdulaziz University, Jeddah, Saudi Arabia; ^6^ Division of Biological and Biomedical Sciences (BBS), College of Health and Life Sciences (CHLS), Hamad Bin Khalifa University (HBKU), Doha, Qatar; ^7^ Nutrition and Bromatology Group, Department of Analytical Chemistry and Food Science, Faculty of Food Science and Technology, University of Vigo, Ourense, Spain

**Keywords:** pharmacophore modeling, virtual screening, molecular docking, molecular dynamics simulation, BRAF, B-Raf

## Abstract

The BRAF gene is responsible for transferring signals from outside of the cell to inside of the nucleus by converting a protein namely B-Raf through the RAS/MAPK pathway. This pathway contribute to cell division, proliferation, migration, and apoptotic cell death of human and animal. Mutation in this gene may cause the development of several cancers, including lung, skin, colon, and neuroblastoma. Currently, a few available drugs are being used that has developed by targeting the BRAF mutated protein, and due to the toxic side effects, patients suffer a lot during their treatment. Therefore this study aimed to identify potentially lead compounds that can target and block the expression of BRAF and subsequently inhibit the cancer. The hits were generated through the pharmacophore model-based virtual screening, molecular docking, pharmacohore model validation, ADME (absorption, distribution, metabolism, and excretion) analysis molecular dynamics (MD) simulation to find more suitable candidate against the overexpress BRAF gene. The pharmacophore based screening initially identified 14 k possible hits from online database which were further screened by ligand scout advance software to get hit compound. Based on molecular docking score of ZINC70454679 (-10.6 kcal/mol), ZINC253500968 (-9.4 kcal/mol), ZINC106887736 (-8.6 kcal/mol), and ZINC107434492 (-8.1 kcal/mol), pharmacophore feature and toxicity evaluation, we selected four possible lead compounds. The dynamic simulation with Schrodinger Maestro software was used to determine the stability of the potential lead candidates with target protein (PDB ID: 5VAM). The results showed that the newly obtained four compounds were more stable than the control ligand (Pub Chem ID: 90408826). The current results showed that the ZINC70454679, ZINC253500968, ZINC106887736, and ZINC107434492 compounds may be able to work against several cancers through targeting the BRAF overexpressed gene. To develop a novel drug candidate, however the evaluation of the web lab based experimental work are necessary to evaluate the efficiency of the each compound against the BRAF target gene.

## 1 Introduction

BRAF also known as the proto oncogene highly responsible for the signal transduction inside the cells for growing the cell number through maintaining the signaling pathway known as MAP/ERK pathway (McCubrey et al., 2007) (Guo et al., 2020). BRAF participates in cell division by activating phosphorylation by binding to Ras-GTP and eventually producing ADP, phosphorylated protein. (Cope et al., 2018). EGF (Epidermal Growth Factor) bind to the cytoplasmic serine and activate the EGFR receptor. In the presence of the two adaptor protein (SOS and GRB2) EGFR knock KRAS to release the GDP. This KRAS allow to bind cystolic BRAF and activate the MEK kinase. Finally through simulating transcription factors contribute in cellular proliferation, differentiation, apoptosis and cell survival (Fanelli et al., 2020).

Genetic mutations of BRAF gene are more common and responsible for developing cardiovascular defects, retardation of mental growth, and also lead to the development of several cancers (A. Richards and Garg, 2010). Mutations in this gene are responsible for more than 80% of skin cancers known as melanomas; others are lung cancer, colon cancer, and also neuroblastoma (Hussain et al., 2015). BRAF mutation in position V600E, which carried about 80% of alteration and V600 K about 10–20%, were responsible for development of cancer in young people, mainly the tumors appear in the parts of body that were not commonly exposed to sunlight (Menzies et al., 2012) (Ascierto et al., 2012) (Luu and Price, 2019). Smokers as well as non-smokers can be radially affected by the cancer, although the cancer in smokers can develop more aggressively and quickly. It has been reported that the BRAF mutation developed in lung adenocarcinoma in people who were never addicted to smoking. The treatment of the lung adenomas is difficult in the case of this mutation as it has been observed as a resistance mutation (Cardarella et al., 2013) (Nguyen-Ngoc et al., 2015). The incidence of colon cancer due to such mutations is higher in females, those over the age of 50, and those with no history of genetically colon cancer. The mutation in chromosome seven from valine to glutamine at position 600 was developed for right-sided colon cancer (Barras, 2015) (Grassi et al., 2021). Both BRAF and KRAF mutations were linked to the development of CRC in two ways: one activated the expression of the KRAS/mTOR/AKT and the other caused instability in cell cycle regulation. (Morkel et al., 2015) (Merz et al., 2021).

The combination target therapy with encorafenib, binimetinib and cetuximab are in the clinical trial phases and showed to more effective rather than the using two drugs (irinotecan + cetuximab) (Roviello et al., 2020) (Geel and Iersel, 2022). One of the aggressive tumors, thyroid cancer, was also developed by the mutation in the BRAF gene. Most of the BRAF mutations occur in the position of the T1799A and others, including the mutation in the K601E in thyroid cancer (Rowe et al., 2007) (Tran et al., 2020). Through examining the total 75 samples, among whom 17 patients developed KRAS mutation and 26 were examined for BRAF mutation, it has been identified that BRAF mutation may lead to developing ovarian cancer in females (Turashvili et al., 2018). Two common mutations were identified, including BRAF in codon 599, and at codon 12 and 13, the KRAS mutation. This mutation is less common (less than 3%) in carcinomas of the stomach, esophagus, and glioma (Ayatollahi et al., 2018).

The BRAF positive mutated patients were under chemotherapy or immunotherapy besides using the targeted therapy. Combination with two drugs (combine therapy) and three drugs (triple therapy) are common in the treatment of BRAF mutations and are also in clinical trial phase (Eroglu and Ribas, 2016) (Patel et al., 2020). Several drugs, such as vemurafenib, dabrafenib, and encorafenib, currently available to treat BRAF mutated cancer based on targeting the mutations V600E and V600K, two types of possible mutations in several cancers. Drugs known as checkpoint inhibitors are being used in triple therapy. Use of these drugs for target therapy has been shown to produce several side effects, including urine in blood, fever, joint pain, skin ulceration, and so on (Proietti et al., 2020) (Tanda et al., 2020). The number of other drugs that can be used during treatment are limited due to drug-drug interactions. Due to their long-time use, most of the BRAF/KRAS mutated tumors are showing resistance to these treatments. A 60 year old female patient was identified the BRAF mutation and treatment with the vemurafenib showed less efficient. A new mutation was also observed after the 11 months of treatment and through the multiple organ failure patient died after 12 months (Wang et al., 2022).

So the development of new drugs with less side effects and also possible to overcome resistance are the first choice for researchers, caused by the BRAF mutations. In our study, we focused on computational drug design to develop more efficient compounds that can be used as drugs through further experiments and validation results. Currently computer based drug discovery are the popular tool for designing a new compounds against the specific target area. For rapid lead compounds identification this pathway follow the pharmacophore modeling, molecular docking, virtual screening, ADMET (absorption, distribution, metabolism, excretion, and toxicity) analysis, molecular dynamics (MD) simulation, and MM-GBSA method (Opo et al., 2021) (Bouback et al., 2021). Molecular docking result usually express the binding possibility between the ligand and receptor, which is the important part for drug efficacy. ADMET analysis by the online database and tool showed the possibility of toxic effect of a lead compound inside the body are more easier to determine rather the conventional method, whereas the toxicity development from the blood sample, stool or urine might create a risk for drug failure (Valasani et al., 2014). As the CADD approach are more convenient, cheap in comparison to the conventional drug design this study aimed to discover lead compound against the BRAF mutations. The identified potentially lead compounds through the in-silico drug design might be able to reduce the BRAF mutated carcinoma.

## 2 Materials and methods

### 2.1 Pharmacophore modelling

To interact with natural molecules, a ligand with a protein structure was retrieved (PDB ID: 5VAM), as well as a three-dimensional structure (Nishiguchi et al., 2017). For identification of the protein structure screening has been performed based on the organism source, X- Ray diffraction method, and refinement resolution also the release date. The attached ligand IC_50_ was already established by several experimental analysis and the toxicity of the attached ligand was low with higher LD_50_ value (2000 mg/kg). PubChem database was used to get the chemical ID of the attached ligand 92J to the target protein (Pub Chem ID: 90408826) (S. Kim et al., 2021). A structure-based pharmacophore model was created using Ligand Scout 4.4.8 advanced software. This powerful software created the interaction between inhibitors and crucial amino acids at the active sites in our target protein. Different pharmacophore properties, such as hydrogen bond donors, charge transfer, hydrophilic and hydrophobic areas, and hydrogen bond acceptors, were used to interpret ligand-receptor interactions. Other parameters such as the quantity of aromatic rings, hybridization state, binding pattern, and receptor molecule distance have been discovered using stepwise algorithms. Using ligand scout, we provided hydrophilic characteristics to the protein to improve the measurement of protein binding. The number of active sites was also measured by using the CASTp (sts.bioe.uic.edu/castp/) software for further analysis (Supplementary Figure S1).

### 2.2 Pharmacophore model verification

A set of active compounds (Supplementary Table S1) were identified from the ChEMBL database (https://www.ebi.ac.uk/chembl/) with an active IC_50_ value (Gaulton et al., 2017). The DUD-E decoy set (obtained from the DUD-E decoys database) was used to evaluate the known active compounds in order to more accurately distinguish between the active and inactive compounds (Mysinger et al., 2012). All the active known compounds and the extracted decoy set were transferred to the ligand scout 4.4.8 advance software to make an “idb” file. Models were generated from the protein-ligand complex through the screening of all active compounds in correspondence of the 4,094 decoy sets. The quality of our selected structure based model was assessed by the AUC value, GH score, and enrichment factor (Wolber and Langer, 2005).

### 2.3 Pharmacophore based virtual screening

A freely assessable database was used to identify the potential lead compounds, including the ZINC Pharmer (http://zincpharmer.csb.pitt.edu/pharmer.html) and ambinter data base (https://www.ambinter.com/#search) (Koes & Camacho, 2012). Both databases were the available source for the determination of the physical and chemical properties such as 2D and 3D structure determination, the boiling point, the melting point, molecular weight, and biological activity of the compounds (Opo et al., 2021). The screening was performed in the Zinc Purchasable database and natural database based on the pharmacophore features generated by the ligand Scout software and previously saved as ‘pml’ file. The chosen compounds had the most similar pharmacophore features to our query compounds. The selected compounds were then subjected to a series of tests, including molecular weight, hydrogen bond donor, hydrogen bond acceptor, and LogP value, all of which were based on Lipinski’s rule of five. All the selected compounds were preserved with their Canonical SMILES ID obtained from PubChem database (https://pubchem.ncbi.nlm.nih.gov/) and proceeded to further study (S. Kim et al., 2021). The database generated from the Zinc and ambinter was validated based on structure based pharmacophore features. A freely accessible ZINC database and also an ambinter database were used to find the most similar compounds. We identified our specific protein structure and a previously prepared library with 14 k compounds was inserted into the Ligand Scout 4.4.8 advance software. The library was screened based on the created pharmacophore features, with the addition of the 1 h bond donor feature. Fitted hit compounds were further subjected to validation based on the relative pharmacophore fit score.

### 2.4 Protein and ligand preparation

The selected protein structures were prepared for docking purposes. The downloaded ‘sdf’ file was opened by the discovery studio and removed the water molecule and also the hetatm. The addition of any necessary bond and deletion of the water molecules was not part of the structural refinement process. The desired protein structure (PDB ID: 5VAM) was obtained and analyzed for the R value-free (0.223), resolution (2.0Å), and observed R-value (0.194). We discovered that a few bonds in the currently selected protein were missing; therefore, we used BIOVA Discovery Studio Tool 16.1.0 to construct a new bond by using the force field (CHARMm). Generally, this force field contribute distinctive effects including electronegativity, stereo electrical effects, polarization, bond stretching and angle bending, on the other hand, are characterized by simple harmonic motion (Hwang et al., 2020).

### 2.5 Grid generation and active site identification

The active site of our protein has been identified and analyzed by the UniProtKB and PrankWeb (https://prankweb.cz/) (Gray et al., 2021) (Jendele et al., 2019). The number of active pockets was also determined using CASTp (CASTp 3.0: Computed Atlas of Surface Topography of Proteins (uic.edu) (Supplementary Figure S1, Supplementary Table S2) (Tian et al., 2018). The presence of hydrogen bonds, lipophilic or hydrophilic interactions, and ionizable charges all affect the protein and ligand’s binding affinity. The PyRx software was used to generate the grid by selecting the active sites of the proteins (Dallakyan and Olson, 2015). The server-generated binding sites were utilized to create a receptor grid box in center with the following coordinates: X = -29.1124, Y = 42.6919, and Z = 8.227 and with the exhaustiveness of 8.

### 2.6 Binding affinity determination by docking

All the selected hit compounds “sdf” files were downloaded from the PubChem database. The compounds and also previously prepared the protein 3D structure were transferred to the PyRx software and docking was conducted by AutoDock Vina. A prominent tool being used in drug design for selecting drugs against various animal diseases and identifying new therapeutic candidates (Dallakyan and Olson, 2015). The compounds were then submitted to the BIOVA Discovery Studio Visualizer Tool 16.1.0 for analysis based on the binding affinity and RMSD value. The validation of the docking has been performed several times with the above mentioned grid generation for the all selected ligands.

### 2.7 ADME profile evaluation

The metabolism and pharmacokinetic properties of a drug are important parameters in determining drug efficacy (Benedetti et al., 2009). Approximately fifty percent of drug candidates fail due to their lack of efficacy and toxicity at the time of the drug development, so the ADME profile analysis is crucial part before drug development. (Opo et al., 2021). Usually elimination of drugs from the body occur through urine and faces, several physiochemical features such as hydrophobicity, lipophilicity, gastrointestinal environment, and blood brain barrier have a direct impact on the ADME profile before elimination of drugs. The bioavailability of a medicine also are being affected by its sex, age, disease state, lipophilicity, hydrophobicity, microbiota, body enzymes, and administration method (Stillhart et al., 2020). For evaluating the ADME profile, such as solubility, GIT absorption, and bioavailability in the case of the ligand, we used the freely available Swiss-ADME server (http://www.swissadme.ch/). Swiss ADME sever is a popular online database for determination of the compound physicochemical and pharmacokinetic properties (Daina et al., 2017).

### 2.8 Evaluation of toxicity

In-silico approaches for analyzing the safety profile of the required chemicals have been developed by computational research (Bouback et al., 2021). Otherwise, these substances could have a negative impact on discovery of new compounds and lead to the failure of drug discovery in the middle of research. The toxicity profile such as hepatic failure, carcinogenicity, immunological response, membrane potential route was easily quantified and qualitatively determined to see the possibility of toxicity before going to the lab based experiment. The computer aided toxicity measurement tools (Toxicity Estimation Software Tool, TEST version 4.2.1) usually commonly used to estimate a chemical’s harmful effect based on its molecular structure. In our study we measured the fathead minnow LC_50_ (96 h), 48-h daphnia magna LC_50_, developmental toxicity, oral rat LD_50_, bioaccumulation factor, and water solubility (at 25°C). Freely access database ProTox-II server (https://tox-new.charite.de/protox II/) was used to detect hepatotoxicity, carcinogenicity, mutagenicity, immunogenicity, and numerous toxicological pathways for selected antagonist (Banerjee et al., 2018).

### 2.9 Protein and ligand preparation for simulation

The simulation of the protein ligand complex tells us the binding pattern and characteristics between the atoms and amino acid residues (Opo et al., 2021). The 100ns dynamic simulation was used to validate our ligand binding to the protein, which had been obtained through the docking studies. The stability of the complex must be assessed to see the possible effect inside the body, as well as the projection of every atom bonding behavior both of ligand and protein molecules during a given time period. Using the Linux command, we conducted our dynamic simulation through utilizing software Schrödinger Release 2020-3 (Academic version) (Bowers et al., 2006). The water model was used to solve the ligand and protein interaction, as well as provide the orthorhombic box shape boundary. By combining the Na+ and Cl-with a 0.15 M salt concentration, the complicated atom buffer box calculation approach was applied. The simulation was run with an ambient temperature of 300 K and a pressure of 1.01325 bar, with a record interval time of 50 ps. The OPLS-2005 force field was used to execute the simulation (Shivakumar et al., 2010).

#### 2.9.1 Trajectory file analysis from ligand protein interaction

The MD simulation’s quality was confirmed, and the simulation scenario was investigated utilizing Schrödinger package’s simulation interaction diagram (SID). The Simulation Interaction Diagram (SID) of the Desmond module was used to evaluate all of the simulation’s data sets (Bowers et al., 2006). Depending on the RMSD, RMSF value, and ligand-protein complex, the simulation trajectory file offered information about the integrity of the protein-ligand interaction complex. The ligand torsion profile has been evaluated to find the rotatable bond were present in the ligand during the simulation trajectories (Jin et al., 2020). Radius of gyration has been used to evaluate the structural compression changes and intra molecular hydrogen bond analysis was performed to identify the presence of internal hydrogen bonds within a ligand molecule.

### 2.9.2 MM-GBSA analysis

A common technique for determining the free binding energy of ligands is the calculation of molecular mechanics with generalized born surface area (MM/GBSA). Typically this analysis based on the receptor ligand complex that are more precise unlike many docking studies grading algorithms and computationally fewer taxing other molecular free energy techniques (Genheden and Ryde, 2015). We estimated the binding free energy of four potentially leads compound and control ligand using the Schrödinger Prime MM/GBSA package (released 2020-3) (Bouback et al., 2021).

## 3 Results

### 3.1 Protein analysis based on pharamacophore features

**FIGURE 1:**
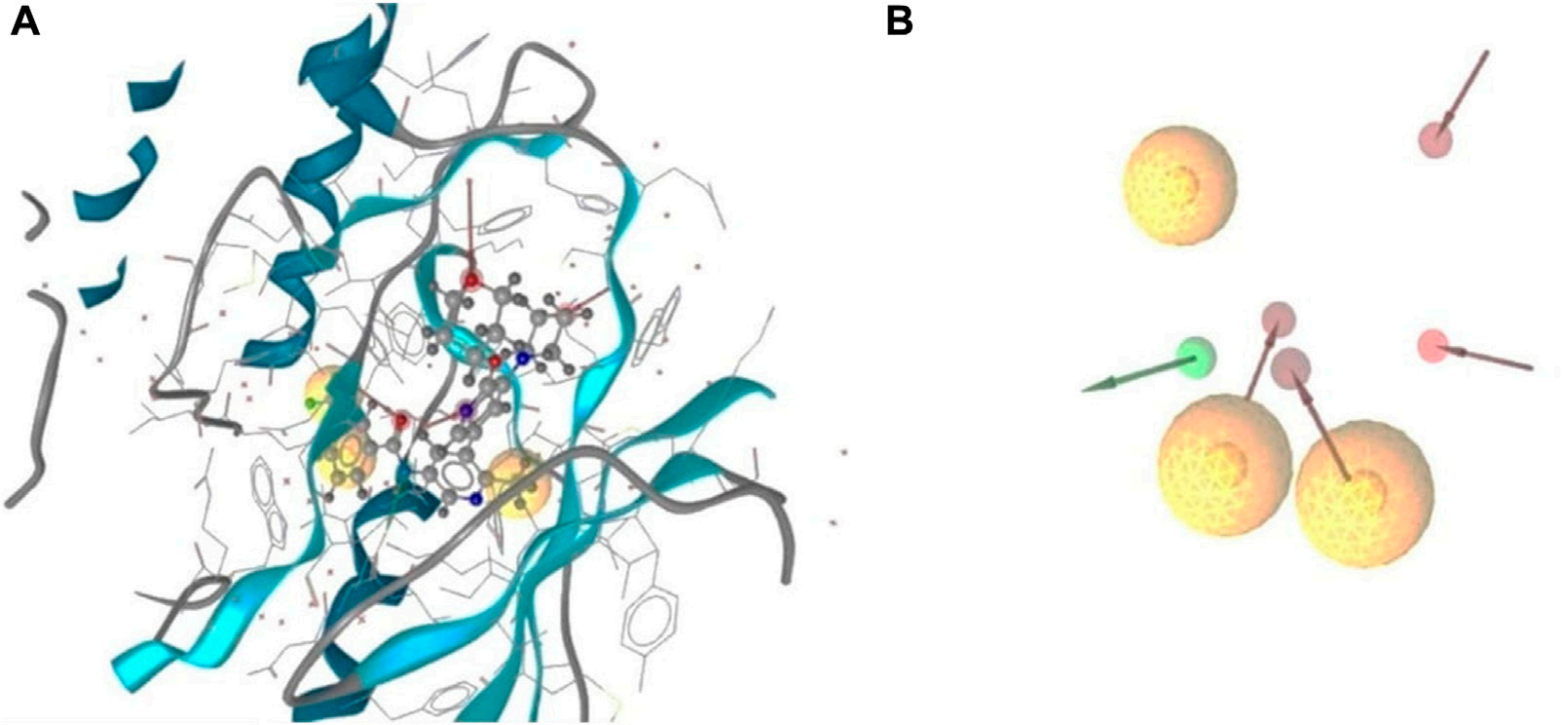
3D structure based on pharmacophore model of BRAF protein ligand complex. Arrangement of the pharmacophore features along with the selected protein structure **(A)**, and the observed pharmacofeatures in the absence of the protein chain **(B)**. Three hydrophobic interactions represented by yellow spheres, red arrows demonstrated H-bond acceptor, and one green arrows depicted the presence of the H- bond donor.

The 3D structure of a protein is important to facilitate the possible drug interaction with the biological activity and is necessary to predict the possibility of efficacy prior to synthesis. The protein was bound to a single ligand, and the structure was determined by x-ray diffraction with a resolution of 2.10, R value free (0.223), R value observed (0.194), and R value work (0.192). The IC_50_ value was calculated from the several assays and was minimum 0.4nM with maximum 1.8 nM and the toxicity of the attached ligand was low with higher LD_50_ value (2000 mg/kg). For determining an active series of inhibitors, it is important to look for enough interaction to attain better biological activity than the current one. The important chemical characteristics were generated using Ligand Scout 4.4.8 advanced critical molecular design software, which was based on a pharmacophore model. Total seven chemical features were observed, including three hydrophobic bonds, three H-bond acceptors, and one H-bond donor without the inclusion of exclusion volume (Figure 1).

Analysis of the interaction with the protein ligand contact indicated the number of hydrophobic interactions were most predominant type of bond among the twelve amino acids. The red arrows represented the interaction of the H-bond acceptors ASP594, HOH917, HOH972, and CYS532. One H-bond donor bond was formed with the GLU501 position of the amino acids (Supplementary Figure S1B).

### 3.2 Pharmacophore model validation

**FIGURE 2:**
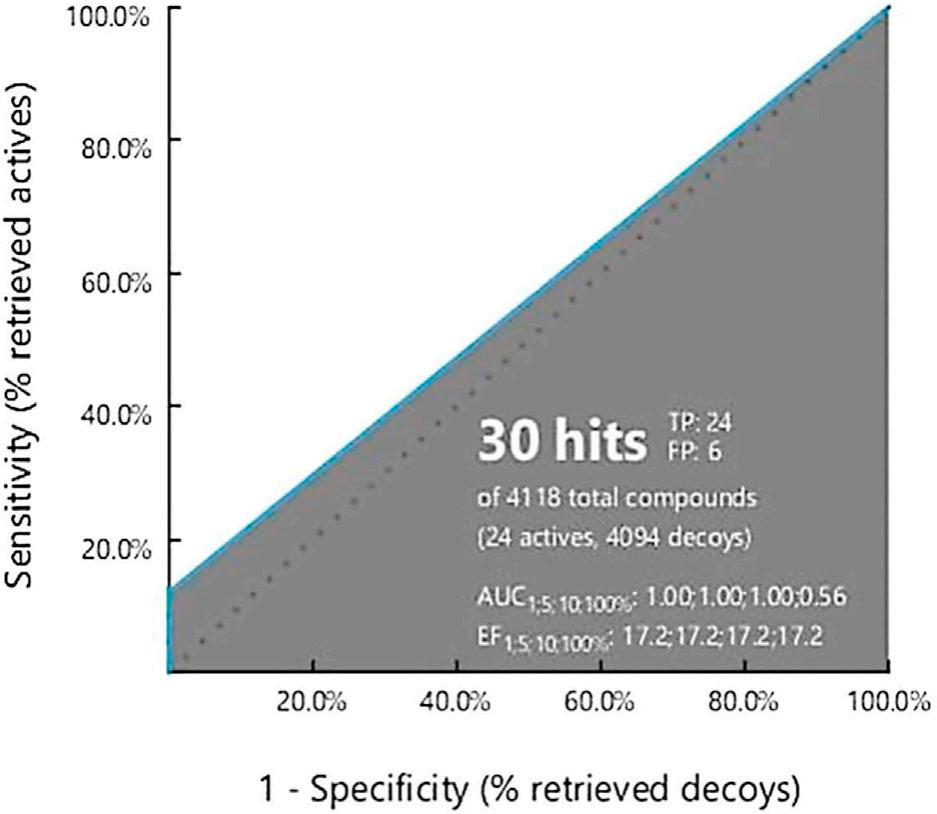
Ligand Scout 4.3 Advance software was used to create a receiver operating characteristic (ROC) curve. The total number of active decoy sets was determined using the dude decoy database’s predefined decoy sets.

Validation is necessary to evaluate the model quality and to obtain an accurate pharmacophore analysis. Validation of the derived pharmacophore model was performed using 24 active known BRAF antagonists in correspondence with 4,094 decoy molecules obtained from the online decoy database. The quality of the curve is represented by the area under the curve (AUC) and the EF value. The early enrichment factor (EF1%) was 17.2, referring to an excellent curve, and the average AUC value was 0.89, indicating good to excellent results (Figure 2).

### 3.3 Dataset generation

The development of data sets is critical for distinguishing the lead compounds. The ZINC and ambinter database are the most commercially available database, with 730 million compounds including natural and chemical compounds, as well as 3D structures and current clinical development conditions (Irwin et al., 2020) (Bouback et al., 2021). The Ligand Scout 4.4.8 advance tool was used to produce pharmacophore features and was submitted to the online database for further screening to identify the potentially active lead compounds. We followed the rule of five in the case of screening the database, the RMSD value was less than or equal to one.

### 3.4 Pharmacophore based virtual screening

Ligand Scout 4.4.8 advanced software was used to create pharmacophore characteristics, which were then transferred to the ZINC database through a ‘mol’ file. We add one H bond features to get the more suitable drug candidate after screening. The search has been completed based on the following rules: The Rule of Five. A total of 155 hits were retrieved when the RMSD value was set to around 1, with a relative pharmacophore fit score of 0.82. The molecules were then docked with the Autodock vina and selected the compounds with the highest binding energy for further investigation and through initially toxicity analysis.

### 3.5 Binding site identification and ligand-protein interaction

**FIGURE 3:**
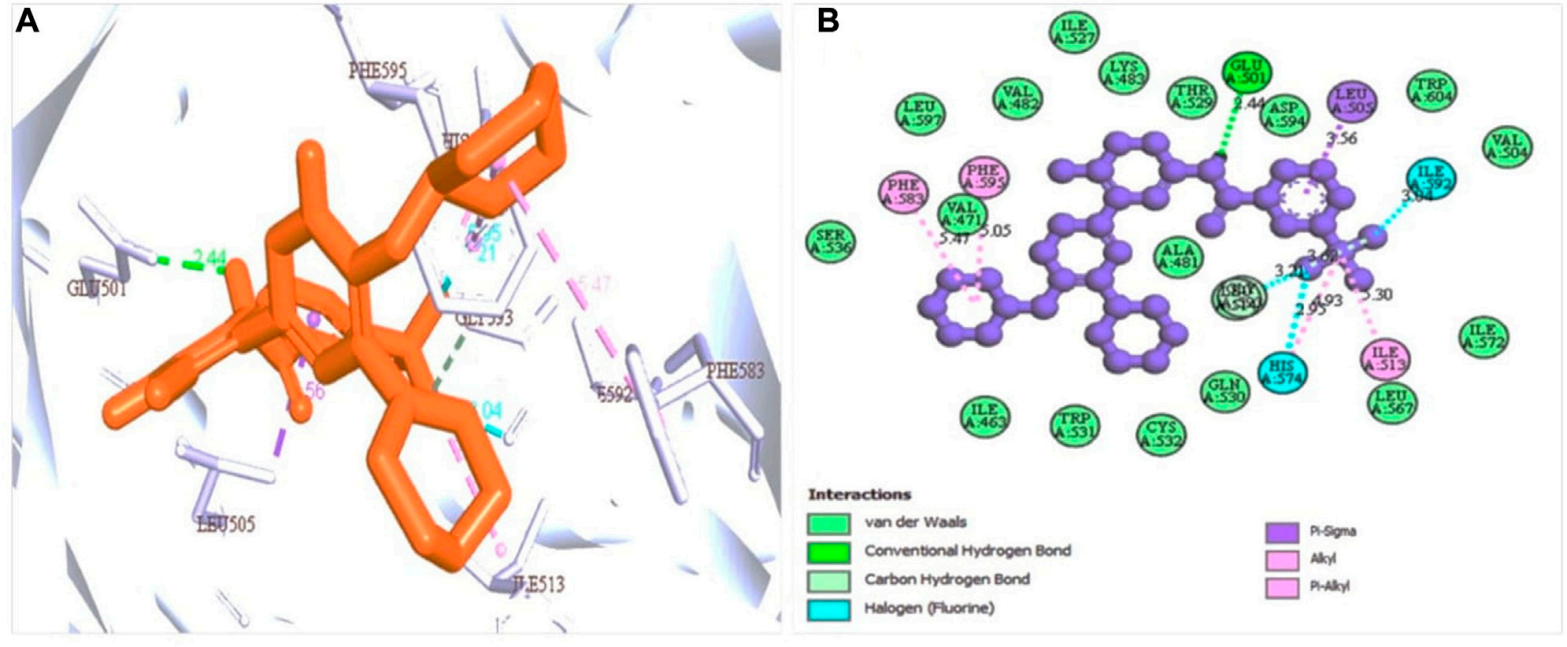
Protein-ligand interaction prediction (BRAF: 5VAM) and the binding site identification. The most predominant type bond was the van der Waals bond and the second more common bond pi-alkyl bond with also the halogen bond. Herein, **(A)** representing the 3D protein-ligand interaction and **(B)** representing 2D interaction of the protein with the ligand.

Based on the structure generated by X-ray crystallography, the selected protein has one attached ligand and separate attachment sites for interacting with the target ligand. A total of seven bond formations with the active sites were observed with multiple amino acid residues indicated by the discovery studio program by analysis of the protein-ligand complex (Figure 3A). The number of active sites has also been determined based on the CASTp software (Supplementary Figure S1A).

### 3.6 Molecular docking

Docking is a technique used in drug development to assess the binding affinity of a protein and its ligand (Salmaso and Moro, 2018). With the addition of one ligand, the BRAF protein was linked to two chains, and we selected the protein through the removal of the water and hetatm. The protein was prepared by combining the force field (CHARMm) and the receptor grid was generated in the PyRx software based on the previously identified active sites (Dallakyan and Olson, 2015). The hits identified through obtained compound library screening as well as the selected known antagonist were sent for docking. The binding affinity score for known antagonist were shown in Supplementary Table S1. For generated hits were selected based on the best binding affinity containing ligands with fewer side effects. These selected potentially lead compounds were considered for further interaction evaluation (Table 1). The docking for the each compound validated to get the exact binding scenario of our selected four compounds, which were showed that all antagonists would be able to bind to the target protein (Supplementary Figure S2).

**TABLE 1:** The binding score generated from the docking with the protein (PDB ID: 5VAM) along together with the compound structure, molecular formula. The compound were selected based on the binding energy and also by evaluating toxicity.

ZINC ID	Compound structure	Binding affinity (kcal/mol)	Molecular formula
**ZINC70454679**	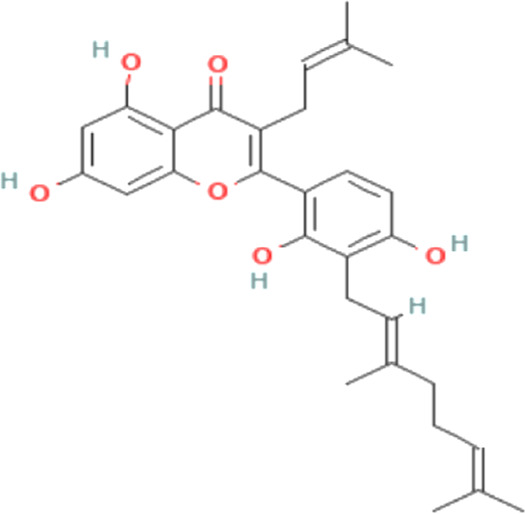	-10.6	C_30_H_34_O_6_
**ZINC253500968**	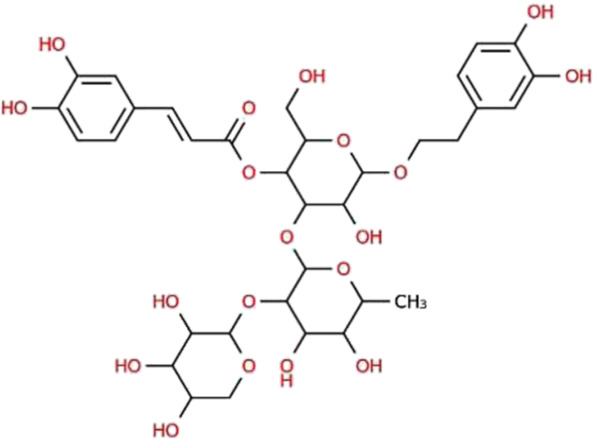	-9.4	C_34_H_44_O_19_
**ZINC106887736**	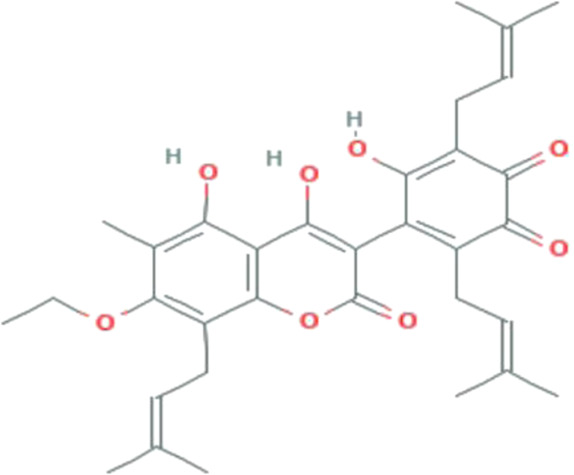	-8.6	C_33_H_38_O_8_
**ZINC107434492**	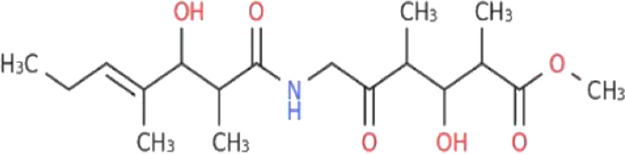	-8.1	C_23_H_32_N_2_O_3_

### 3.7 Identification of the protein-ligand interaction

**FIGURE 4:**
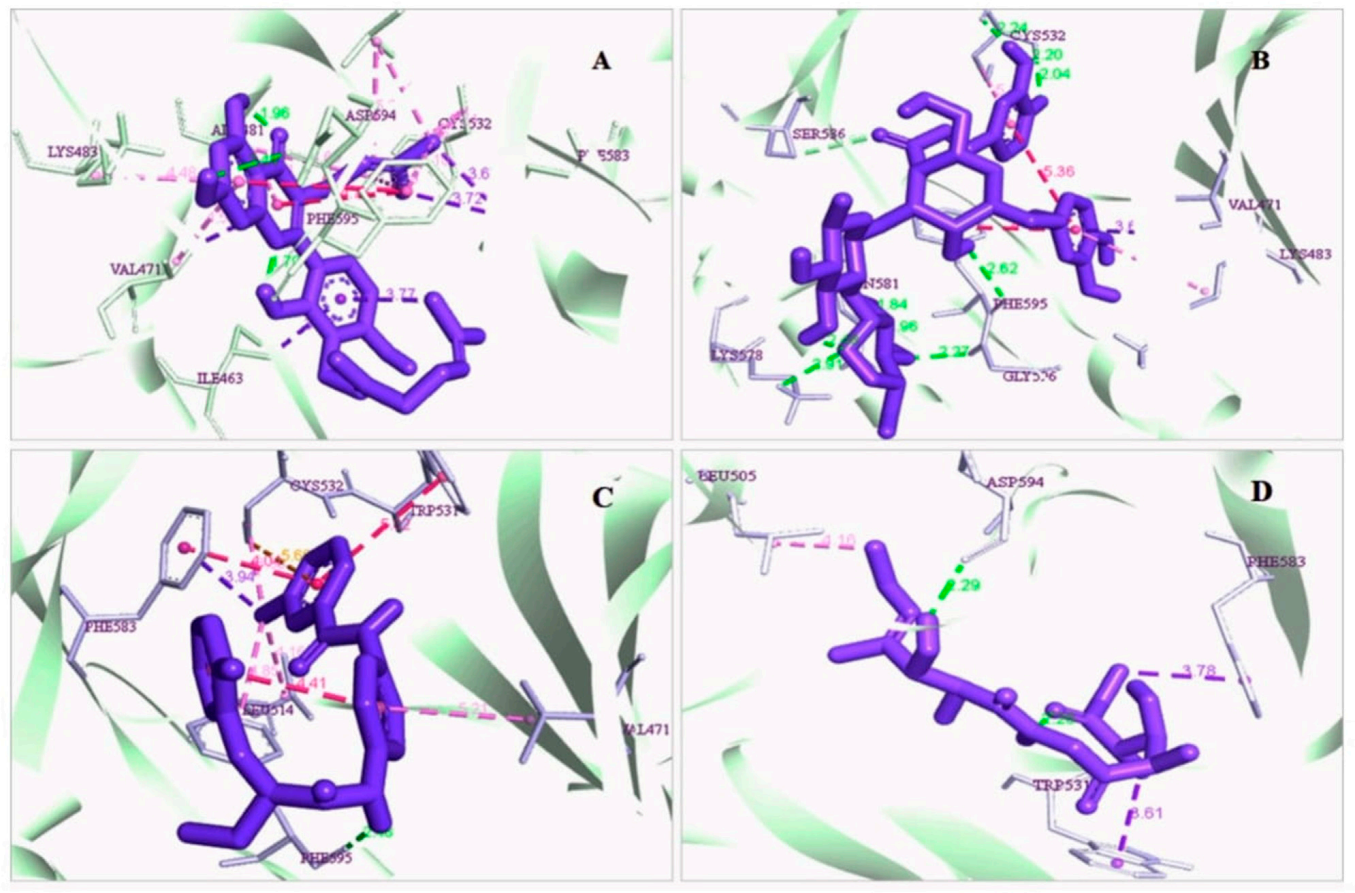
3D interaction of the selected antagonist with the protein complex (PDB ID: 5VAM). Our ligands **(A)** ZINC70454679, **(B)** ZINC253500968, **(C)** ZINC106887736, and **(D)** ZINC107434492 shown the better interaction with the 5VAM protein. Based on the binding affinity score and also the toxicity analysis, four compounds were selected.

**FIGURE 5:**
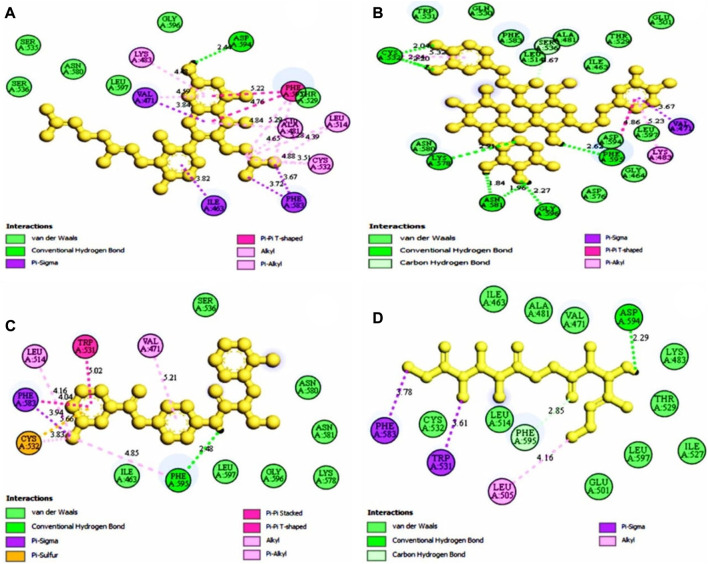
The selected antagonist’s 2D interaction with the protein complex (PDB ID: 5VAM). Our ligands **(A)** ZINC70454679, **(B)** ZINC253500968 **(C)** ZINC106887736, and **(D)** ZINC107434492 had the best protein interaction. Four compounds were chosen based on the docking score as well as the toxicity analysis.

The protein-ligand interaction is important to observe the possibility of achieving better biological functions (Opo et al., 2021). In our experiment, we discovered that the higher the binding affinity, the greater the interaction with the amino acids’ various targets. In the interaction analysis, ZINC70454679 showed the formation of six bonds with the various amino acids, such as six van der Waals bonds (SER536, SER535, ASN580, LEU597, GLY596, THR529), one conventional hydrogen bond (ASP594), five pi-sigma bonds (ILE463, VAL471, PHE583), pi-pi T shaped (PHE595), three alkyl bonds (ALA481, LEU514, CYS532), and four pi-alkyl bonds (PHE595, ALA481, VAL471, LYS483). In ZINC253500968, seven conventional hydrogen bonds were formed and interacted with GLY596, ASN581, CYS532, PHE595, one carbon hydrogen bond (SER536), one Pi-Sigma bond (VAL471), one Pi-Pi T-shaped bond (PHE595), and two Pi-Alkyl bonds (CYS532, LYS483), but the maximum amino acids showed van der Waals bonds (TRP531, GLN530, PHE583, LEU514, ALA481, ILE463, THR529, GLU501, ASN580, ASP536, GLY464, ASP594, LEU597). ZINC106887736 has been shown to interact with several amino acids such as van der Waals bonds (SER536, ASN580, ASN581, LYS578, GLY596, LEU597), conventional hydrogen bonds (PHE595), Pi-Sigma (PHE583), Pi- Sulfur (CYS532), Pi-Pi Stacked (PHE583), Pi-Pi T-shaped (TRP531), two alkyl bonds (LEU514, CYS532), and Pi-alkyl bonds (VAL471). ZINC107434492, on the other hand, formed a van der Waals bond with ten amino acid residues (LEU514, ILE463, ALA481, VAL471, LYS483, LEU597, THR529, GLY596, GLU501, ILE527, CYS532), one conventional hydrogen bond (ASP594), one carbon hydrogen bond (PHE595), two Pi-Sigma bonds (TRP531, PHE583) and one alkyl bond (LEU505) with the BRAF protein (Figure 4 and Figure 5).

### 3.8 Pharmacophore features analysis

**FIGURE 6:**
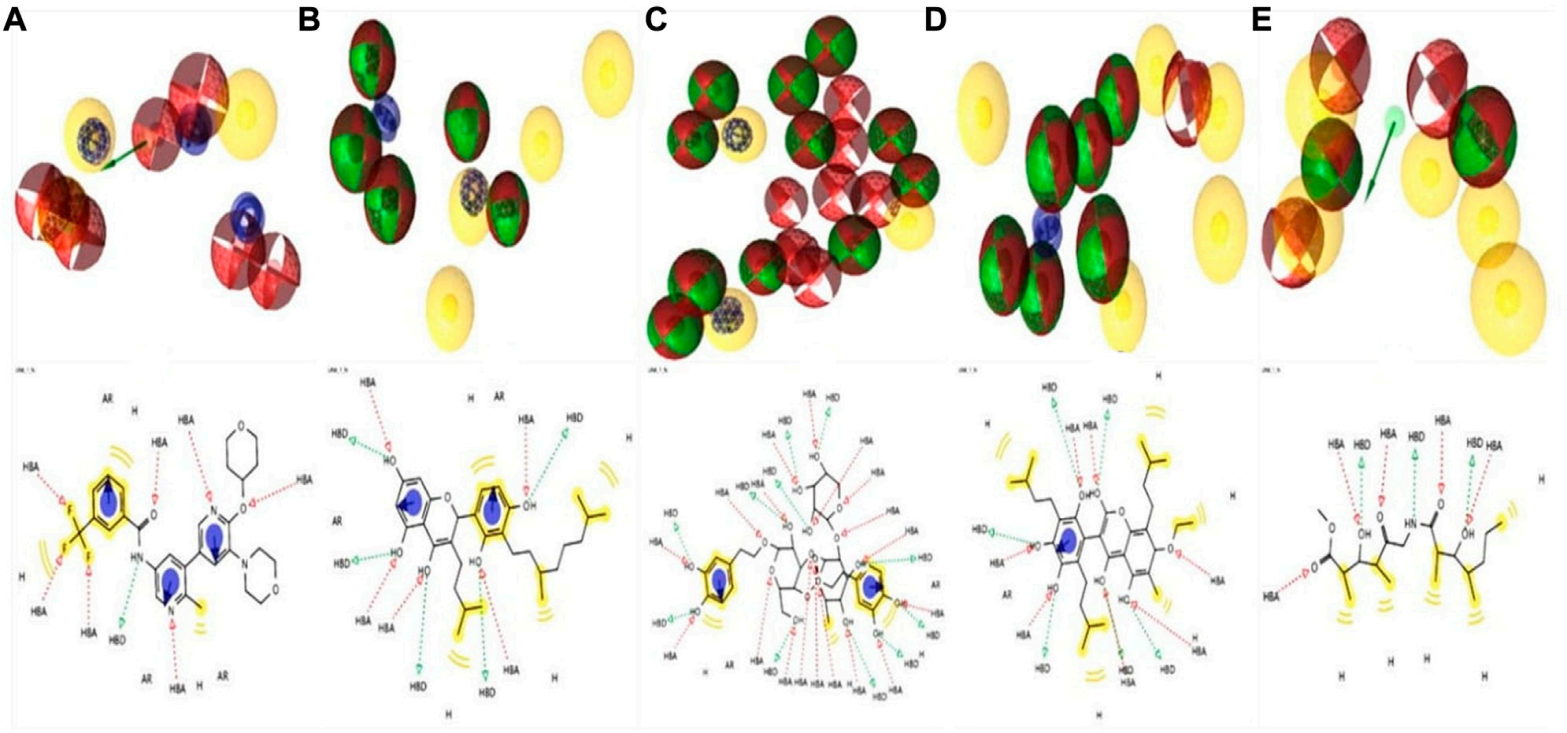
Analysis of four compounds with the target BRAF protein using 2D and 3D pharmacophore characteristics. The pharmacophore features of the **(A)** ligand (Pub Chem ID 90408826) coupled to the protein (PDB ID: 5VAM) were less than the **(B)** ZINC70454679, **(C)** ZINC253500968, **(D)** ZINC106887736, and **(E)** ZINC107434492 our selected four antagonist.

Lead development screening is an important aspect of the biopharmaceutical industry prior to the development of a medication, and these features predict the possibility of binding with the macromolecule. The analysis of pharmacophore features predicts the H, AR, HBA or HBD, PI, and NI characteristics among the compounds, which are essential parts of predicting binding capacity among the proteins (Batool et al., 2019). By using the rule of five, we were able to interpret the drug-likeness and non-drug aspects of the top four higher binding energy molecules: ZINC70454679, ZINC253500968, ZINC106887736, and ZINC107434492. The pharmacophore characteristics generated by the examined ligands were superior to or comparable to the antagonist attached to the protein (PDB ID: 5VAM) (Figure 6).

### 3.9 Pharmacokinetic (ADME) evaluation

For computational drug design, it enabled us to get the absorption, distribution, metabolism, excretion, and toxicity analysis before going to establish a molecule as a drug candidate. From administration to excretion by sweat, urine, or stool, the key pathways for a drug showing efficacy inside the body are absorption, distribution, metabolism, and excretion (Watanabe et al., 2019). For higher bioavailability, the drug’s volume of distribution to the tissue and target site must be increased, and to lessen side effects and toxic effects, the drug should be washed out easily through following the metabolic pathway. We evaluated ADME properties such as lipophilicity, water-solubility, drug-likeness, and medicinal chemistry by using the online Swiss ADME database (http://www.swissadme.ch/) (Daina et al., 2014). The characteristics of the drugs provide us with important information regarding the formulation (tablet, ointment, capsule, injection, and inhaler) and its route of administration (Table 2).

**TABLE 2:** Different features of the four selected compounds we chose were identified. The table depicts the several physical, chemical, pharmacokinetic, and drug likeness aspects.

Properties	Parameters	ZINC70454679	ZINC253500968	ZINC106887736	ZINC107434492
**Physico-chemical properties**	MW (g/mol)	490.59	756.70	562.65	357.44
	Heavy atoms	36	53	41	25
	Arom. heavy atoms	16	12	10	0
	Rotatable bonds	8	13	9	12
	H-bond acceptors	5	19	8	6
	H-bond donors	4	11	3	3
	Molar Refractivity	147.01	174.81	162.34	94.98
**Lipophilicity**	Log P_o/w_	4.52	2.95	4.94	2.58
**Water Solubility**	Log S (ESOL)	Poor	Soluble	poor	Soluble
**Pharmacokinetics**	GI absorption	Low	Low	Low	High
	CYP3A4 inhibitor	No	Yes	Yes	No
	BBB permeant	No	No	No	No
**Drug likeness**	Lipinski, Violation	Yes	Yes, 3	Yes, 1	Yes
	Bioavailability Score	0.55	0.17	0.56	0.55
**Medi. Chemistry**	Synthetic accessibility	4.78	7.28	5.27	4.40

### 3.10 Toxicity prediction

Because of its accuracy, efficiency, and availability for both synthetic and natural chemicals, toxicity analysis is a common technique to choose a suitable therapeutic candidate using computer-based drug discovery. TEST and ProTox-II are two free tools that can be used to test a compound’s toxicity. The drug candidate must be chosen based on toxicity, as the less toxic drugs are better for disease intervention. Table 3 showed the results of the cytotoxicity, mutagenicity, carcinogenicity, hepatotoxicity, and LD_50_ (mg/kg) tests based on software analysis. Three compounds, such as ZINC70454679, ZINC253500968, and ZINC106887736, were shown to have immunologic reactions, except ZINC107434492. Other toxicity data was not available for these compounds, although some data was missing in the case of ZINC106887736.

**TABLE 3:** Various toxicities (Organ Toxicity, Toxicity Class, Tox21-Nuclear receptor signaling pathways, Tox21-Stress response pathway, Fathead minnow LC50 (96 h), Developmental toxicity, Water solubility, Oral rat LD50, and Bioaccumulation factor of selected four compounds) were investigated.

Endpoint	Target	ZINC70454679	ZINC253500968	ZINC106887736	ZINC107434492
**Organ Toxicity**	Hepatotoxicity	Inactive	Inactive	Inactive	Inactive
**Toxicity Endpoints**	Carcinogenicity	Inactive	Inactive	Inactive	Inactive
	Immunotoxicity	Active	Active	Active	Inactive
	Mutagenicity	Inactive	Inactive	Inactive	Inactive
	Cytotoxicity	Inactive	Inactive	Inactive	Inactive
	LD_50_ (mg/kg)	159	5,000	300	8,300
	Toxicity Class	3	5	3	6
**Tox21-Nuclear receptor signaling pathways**	Androgen Receptor (AR)	Inactive	Inactive	Inactive	Inactive
	Aryl hydrocarbon Receptor (AhR)	Inactive	Inactive	Inactive	Inactive
**Tox21-Stress response pathway**	Heat shock factor response element	Inactive	Inactive	Inactive	Inactive
	Mitochondrial Membrane Potential (MMP)	Inactive	Active	Active	Inactive
	Phosphoprotein (Tumor Supressor) p53	Inactive	Inactive	Inactive	Inactive
**Fathead minnow LC50 (96 h)**	mg/L	267.57	N/A	N/A	18.40
**48-h *Daphnia magna* LC**_50_	mg/L	11.58	149.64	N/A	85.90
**Developmental toxicity**	value	1.19	N/A	N/A	0.72
**Oral rat LD**_**50**_	mg/kg	151.54	N/A	N/A	124.33
**Mutagenicity**	Result	Negative	Negative	N/A	Negative
**Water Solubility (25°C)**	mg/L (predicted Value)	489.55	6,263.21	N/A	805.91

### 3.11 Protein ligand complex structure analysis

The interaction between the protein and ligand with the same environmental factors inside the human body is predicted by molecular simulation. It also tells us how many different sorts of bonds there are and how they interact with the different amino acids throughout time. The concentration of the ion, pH all were kept near to the same environment of human body before proceeding to simulation. The ‘pdb’ files of the compounds were chosen for simulation based on the binding score generated by docking. The protein secondary structure elements were analyzed in each trajectory frame at the time of simulation (Supplementary Figure S3).

#### 3.11.1 Analysis of the protein RMSD

**FIGURE 7:**
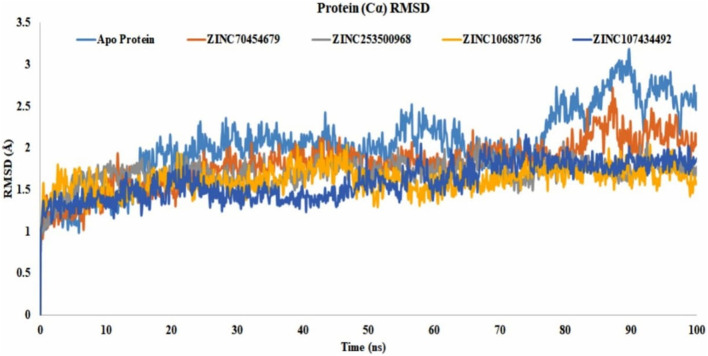
Protein RMSD value of all selected compounds ZINC70454679 (gray), ZINC253500968 (orange), ZINC106887736 (blue), and ZINC107434492 (green). The Apo–Protein has been shown by the light blue color and control ligand CID 90408826 indicated through gold color.

The RMSD value showed us the number of atoms that were not fitted properly. Most of the compounds have shown that they were stable with the interaction between the protein and ligands. The values of more than 3Å indicated the conformational changes of the protein and the system were unstable. The analysis of all selected proteins ZINC70454679, ZINC253500968, ZINC106887736, and ZINC107434492 revealed that most of the 100ns are stable, with the exception of the apo protein, which fluctuated at 89.6ns and again at 90.5ns. The selected compound has shown lower fluctuations (Figure 7) in contrast to the control protein (5VAM).

#### 3.11.2 Ligand RMSD analysis

**FIGURE 8:**
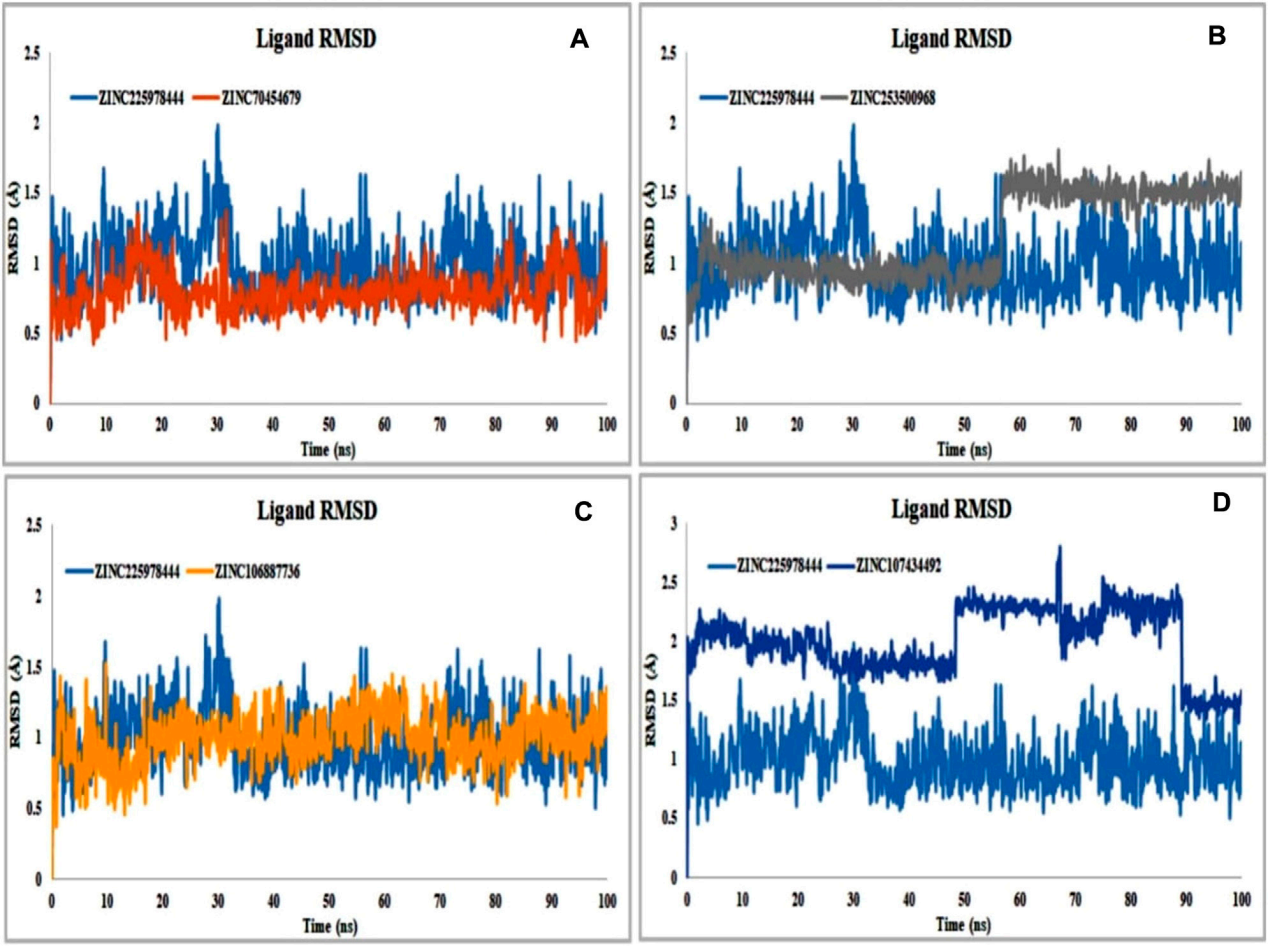
Protein compatibility-RMSD value determined from the ligand interaction. Several colors such as **(A)** ZINC70454679 (gold), **(B)** ZINC253500968 (grey), **(C)** ZINC106887736 (orange), and **(D)** ZINC107434492 (blue) indicate the number of ligands and their expression patterns in comparison to control ligand (CID: 90408826).

Binding of the ligand with the protein and their stability is the important parameters for the proper efficacy of a drugs. The selected compound ZINC225978444 was found to be the most unstable in the interaction with the protein-ligand complex in our experiment. In 49.2ns it showed instability and again was stable until 66.6ns and again unstable from 66.2 to 68.7ns. Finally, it comes to the stability of the 89.5ns through slight unstability at 88.7ns. In 56.6ns, the compound ZINC253500968 showed slight unstability and again came to stable 57.3ns. On the other hand, all other compounds showed good stability within the protein-ligand interaction complex (Figure 8).

#### 3.11.3 RMSF analysis

**FIGURE 9:**
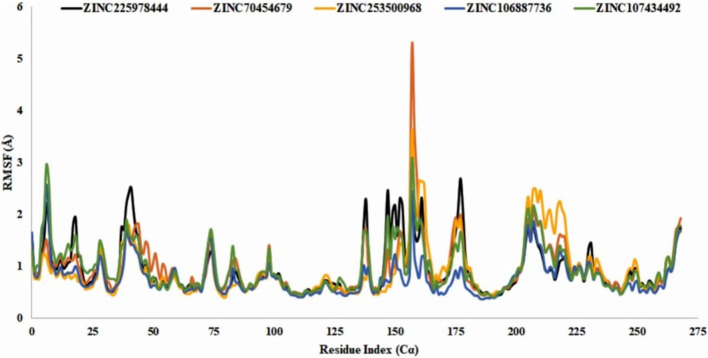
RMSF value identification of all the selected compounds from the obtained Cα value. The color of the graph indicated the compounds RMSF value such as control ligand CID 90408826 (black)**,** ZINC70454679 (orange), ZINC253500968 (gold), ZINC106887736 (blue), and ZINC107434492 (green). N- and C-terminal showed fluctuation more than the other but the value with the 3Å.

RMSF analysis showed that the local conformational changes in the protein and the compounds were used as antagonists. The local fluctuations of the protein with the interaction of our selected compounds were determined by the Cα residue index (Figure 9). In our experiment, all the selected compounds, CID 90408826 (BRAF: 5VAM), ZINC253500968, ZINC106887736, and ZINC107434492, showed a stable RMSF value within the 1-3Å except ZINC70454679, which showed a little fluctuation at position 157 amino acid residue (PHE610). ZINC253500968 showed a slight fluctuation at the same amino acid position of 157 and then came to a stable position again.

#### 3.11.4 Identification of protein-ligand interaction

**FIGURE 10:**
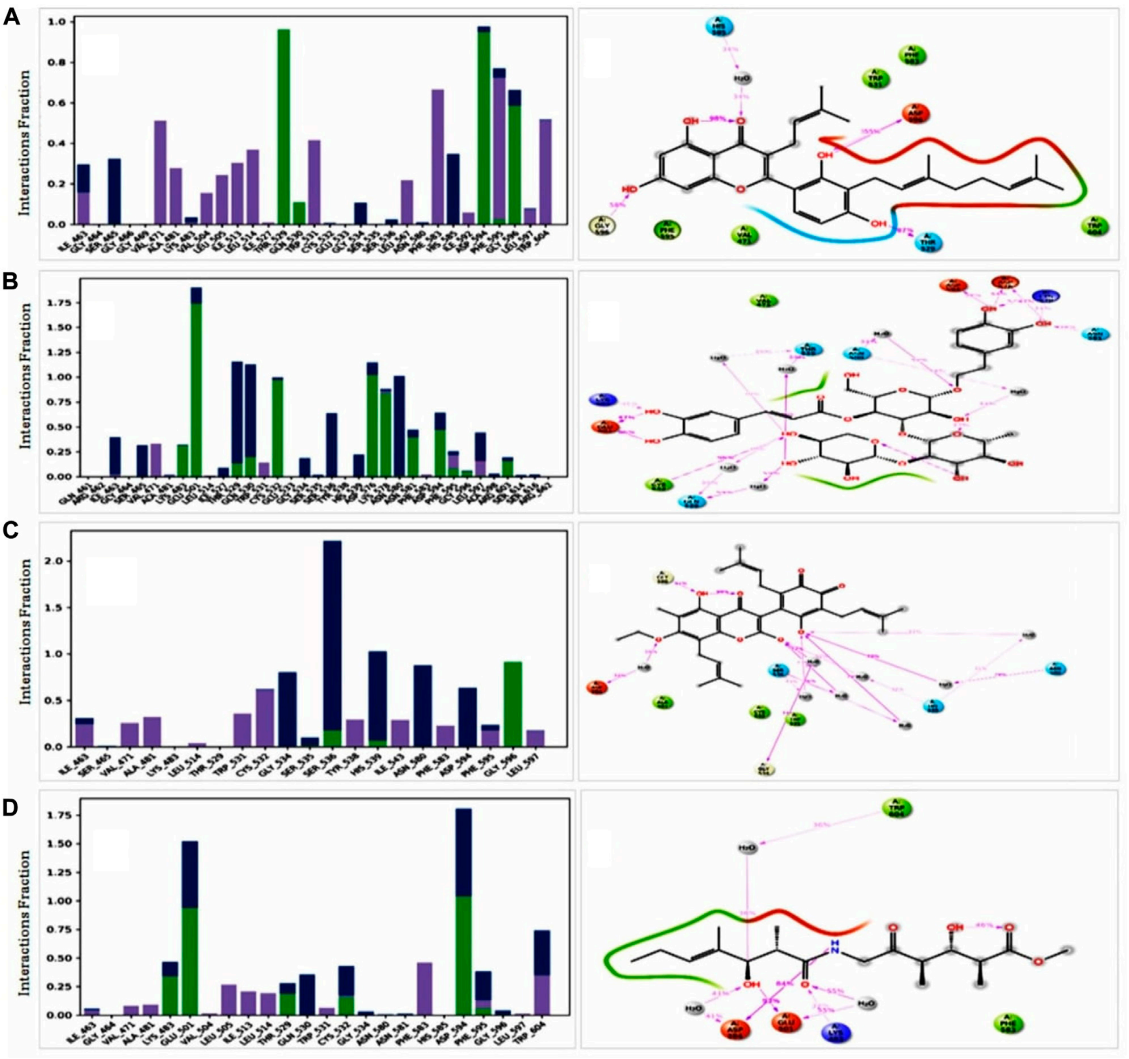
Protein ligand interaction of among the selected compounds by histogram and 2 days summary. All compounds **(A)** ZINC70454679, **(B)** ZINC253500968, **(C)** ZINC106887736, and **(D)** ZINC107434492 shown better contact with the protein. Several colors indicated the bond types such as hydrogen bond (green), hydrophobic (gray), ionic (red) and water bridges (blue), negative charge (gold).

For consideration of a compound as a drug molecule, it should have the properties to bind with the target protein by several bonds, such as conventional hydrogen bonds, hydrophobic, hydrophilic interactions, pi-sigma interactions, pi-sigma bonds, etc (Varma et al., 2010). The majority of the amino acid residues in all compounds came into contact with the ligands during the various interactions. In ZINC70454679, three amino acids did not come into contact, such as GLY466, GLU533, SER535 and six amino acid residues (GLN461, ARG462, GLU533, TYR538, and ARG662) did not bind with the protein in the case of ZINC253500968 (Figure 10).

**FIGURE 11:**
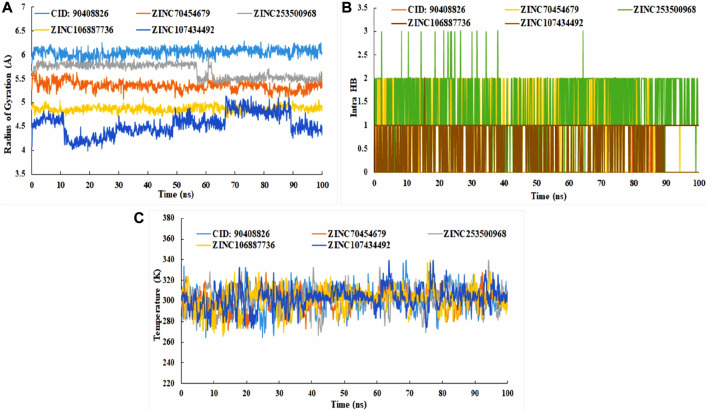
Radius of gyration **(A)**, intra molecular hydrogen bond **(B)**, and temperature changes **(C)** for the protein-ligand complex during the 100ns dynamic simulation.

To comprehend how the selected four antagonists’ structural evolution were changed across the simulation trajectories analysis from 0 to 100ns, the torsional conformations of each rotatable bond in the ligand were determined (Supplementary Figure S4). Gyration analysis showed that the all the compounds were compressed throughout the simulation time except ZINC253500968. Structural transformation occurred from 10 to 40ns as sudden dropped was observed for the ZINC253500968. In case of other compounds sharp, sudden dropped and peak were not observed, which indicated the low structural change (Figure 11A). The number of the intra molecular hydrogen bond was present overall compounds and the higher in ZINC253500968 (Figure 11B). The temperature variations has been mentioned during the simulation and the showed the fluctuations was low during 100ns simulation time (Figure 11C).

#### 3.11.5 MM-GBSA analysis

**FIGURE 12:**
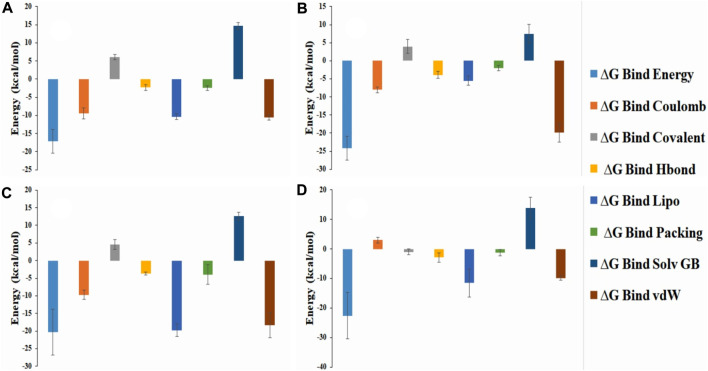
Representation of the several energy components of ligands and net MM-GBSA binding free energy from the protein and selected potentially lead compounds i.e., **(A)** ZINC70454679 **(B)** ZINC253500968 **(C)** ZINC106887736 and **(D)** ZINC107434492.

Usually MM/GBSA analysis are being used to determine the binding free energy of the selected anatomist from the protein-ligand complex from the trajectory simulation file. Analysis of the free binding energy in our selected compounds such as ZINC70454679, ZINC253500968, ZINC106887736, and ZINC107434492 showed the higher net negative binding energy free value (Figure 12). The complex analysis showed the binding energy -18.12 kcal/mol, -24.17 kcal/mol, -20.30 kcal/mol, -22.64 kcal/mol respectively for ZINC70454679, ZINC253500968, ZINC106887736, and ZINC107434492. The result depicted that all four potentially lead compounds maintained good interaction with the protein complex. At the same time screening, physical and chemical components of our selected ligands were indicated a significant contribution of coulomb energy and Van Der wall interaction energy.

## 4 Discussion

BRAF mutation in the metastatic colorectal cancer showed poor chemotherapeutic response and shorter the survival rate for patients. V600E mutation in BRAF overexpressed carcinoma consist of near about 80% and other 20% remain in V600 K. Activation of the mitogen-activated protein kinase pathway are responsible for accelerating the RAF (Rapidly accelerated Fibro sarcoma and conduct signal to the signal regulated kinase (MEK), and finally participate cell proliferation and survival through activating the ERK kinase (Extra cellular signal Regulated Kinase) (Leonetti et al., 2018). Holderfield et al. (2014). It has been identified BRAF mutations as the most frequent mutations related to human carcinomas such as thyroid cancer, ovarian cancer, hepatic carcinoma, and hairy cell leukaemia. The most common mutation has been observed at V600E by sequencing (Yan et al., 2022). The discovery against this BRAF mutation target are in some clinical trial phase and currently using the drugs showing the side effects after administration to the patients (A. Kim and Cohen, 2016) (Holderfield et al., 2014). However, no drugs are available with fewer side effects and to cure cancer as well. Therefore, our study aim was to find potentially lead compounds through computer based drug design that would be effective against the overexpression of the BRAF protein. For computer aided drug design, the BRAF protein structure identified from the online protein database screening and selected protein based on the resolution, R-value free and R-value observed (Ormö et al., 1996). The ligand attached to the protein were also evaluated by the toxicity software Swiss ADME and also by the ProTox II database (Daina et al., 2017) (Opo et al., 2021) (Rella et al., 2006). The active antagonists were currently available on the market, as well as the literature search was considered for the virtual screening, molecular docking, and also the comparison with the selected compounds. The ZINC and Ambinter databases were further screened for getting the natural compounds with the generation of the pharmacophore features from the Ligand Scout 4.4.8 advanced software (Wolber and Langer, 2005). We arranged all the structures for antagonists with their IC_50_ values and further generated the ROC curve from the ligand scout software, and our obtained ROC curve indicated the satisfactory identification capability. The obtained compounds were docked with the PyRx tool, and compounds were selected based on the docking results (Dallakyan and Olson, 2015).

**FIGURE 13:**
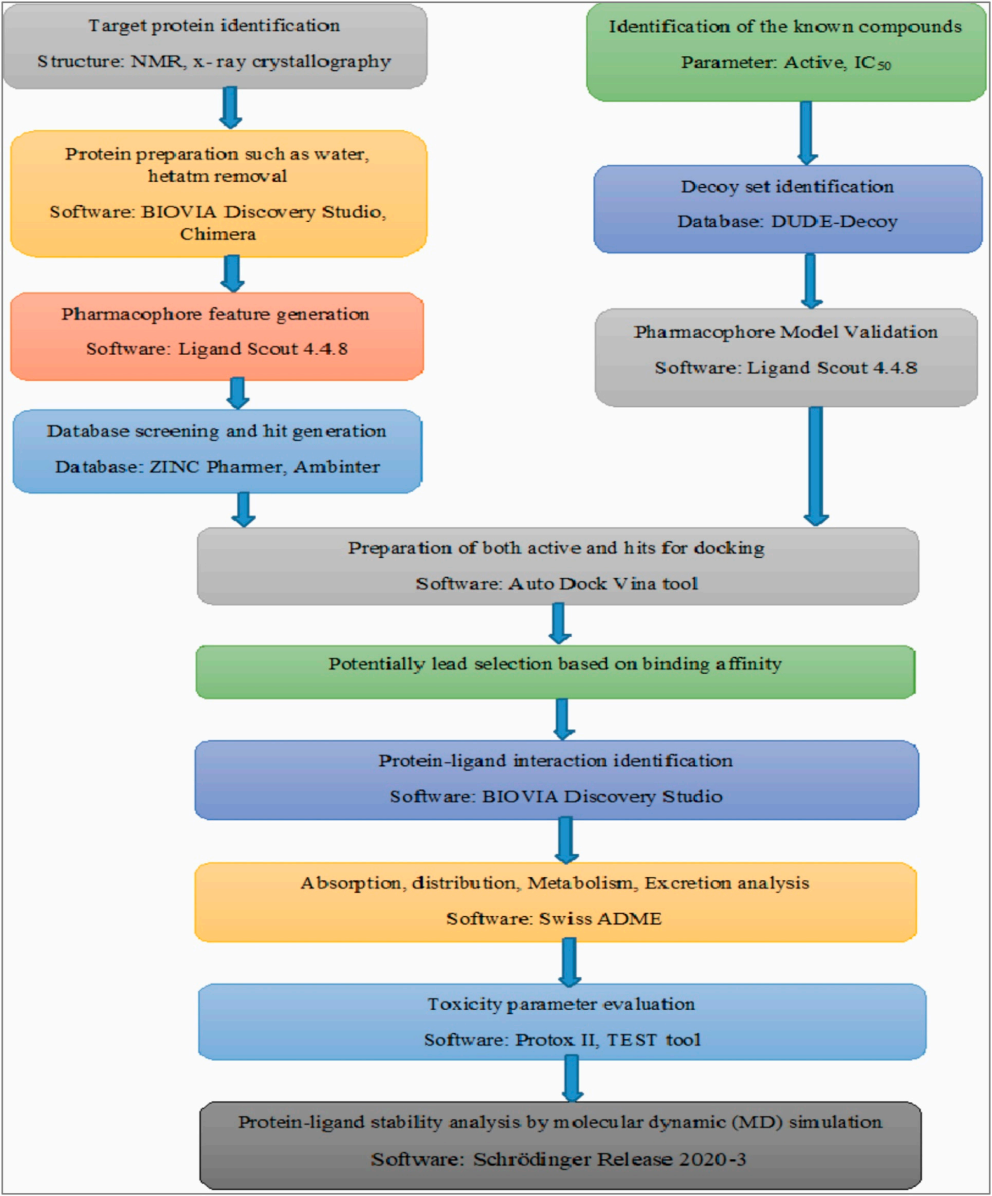
Overall the workflow in our computer aided drug design. The figure has mentioned from the beginning of the starting of the protein selection, virtual screening, protein-ligand interaction and stability analysis.

All the selected four compounds in our in-silico drug design, PubChem ID: 90408826, ZINC253500968, ZINC106887736, and ZINC107434492, indicted the least toxicity based on the evaluation of the ADME profile. Although immunotoxicity is more common in the cases of control ligand (PubChem ID: 90408826), ZINC253500968, and ZINC106887736, the ADME profiling of ZINC107434492 revealed no toxicity. The compound ZINC253500968 violated three of the five Lipinski rules but was not harmful to humans or animals due to its low toxicity. For the further protein ligand complex stability evaluation of lead compounds, we used molecular dynamic simulation for 100 ns. The trajectory files obtained from the simulation were analyzed based on the RMSD, RMSF value, protein–ligand interaction, intra molecular hydrogen bond, radius of gyration, ligand torsion profile were been evaluated and showed the stability of our four lead compounds. As our potentially lead drug candidates having lower toxicities profile so it could be provided an opportunity to develop lower toxic drug for the researcher and possible to treat BRAF overexpression related cancer. The overall workflow by the in-silico drug design has been mentioned in Figure 13, from the starting of the selection of protein, selected antagonist and molecular dynamic simulation analysis. The majority of the patients were identified as having mutations in BRAF-V600E and were most predominant in thyroid carcinoma, colon cancer, and skin cancer (Tufano et al., 2012) (Lasota et al., 2014) (Ascierto et al., 2012). As a result, the development of a BRAF antagonist will alter treatment options in cancer treatment from the early to late-stage carcinoma and may aid in overcoming drug resistance.

## 5 Conclusion

In this study, four identified compounds were selected ZINC70454679, ZINC253500968, ZINC106887736, and ZINC107434492 through the virtual screening as a potential lead candidates for BRAF protein overexpression related carcinoma. It may be able to increase apoptosis in several cancer cells by targeting the BRAF protein. The higher binding affinity with the protein showed the docking score from -8.1 to -10.6 kcal/mol and have higher possibilities to bind the target area. The stability of the protein and four ligand complexes were validated through using the dynamic simulation and trajectory file analysis indicated the four key amino acid residues i.e., PHE583, CYS532, VAL471, LEU597, ILE463 based on the interactions. The binding energy was calculated based on the MM-GBSA method and predicted that the lower binding energy due to more stable hydrogen bonds among the protein-ligand complex. Based on the evaluation ADME and toxicity profile of potentially lead compounds, they have lower toxic effects and ZINC107434492 is the most suitable candidate for further analysis as it had no toxicity. Evaluating the in-silico toxicity profile of the other available marketed drugs against the BRAF overexpression cancer such as sorafeniib, TAK-632 our selected antagonist would have the more possibility to reduce the side effects currently possible anti-cancer treatments. The use of virtual screening, molecular docking, pharmacophore model validation, ADMET profile analysis, protein-ligand binding analysis by discovery studio, and dynamic simulation revealed that these compounds should go for further *in-vitro* as well as *in-vivo* work, which may be able to discover new BRAF antagonists.

## Data Availability

The datasets presented in this study can be found in online repositories. The names of the repository/repositories and accession number(s) can be found in the article/Supplementary Material.

## Abbreviations

BRAF, v-raf murine sarcoma viral oncogene homolog B1; AUC, Area under curve; CAAD, Computer Aided Drug Discovery; CRC, Colorectal cancer; EGFR, Epidermal Growth Factor Receptor; IC_50_, Inhibitory concentration; IDB, Intermediate Debug File; KRAS, Kirsten rat sarcoma viral oncogene homolog; LC50, Lethal Concentration 50; LD_50_, Lethal Dose 50; MOL; MM-GBSA, Molecular mechanics with generalised Born and surface area solvation; MAPK, mitogen-activated protein kinase; SDF, Structure data file.

## References

[B1] Ascierto P. A. Kirkwood J. M. Grob J. J. Simeone E. Grimaldi A. M. Maio M. (2012). The role of BRAF V600 mutation in melanoma. J. Transl. Med. 10 (1), 85. 10.1186/1479-5876-10-85 22554099 PMC3391993

[B2] Ayatollahi H. Tavassoli A. Jafarian A. H. Alavi A. Shakeri S. Shams S. F. (2018). KRAS codon 12 and 13 mutations in gastric cancer in the northeast Iran. Iran. J. Pathol. 13 (2), 167–172. 10.30699/ijp.13.2.167 30697286 PMC6339504

[B3] Banerjee P. Dehnbostel F. O. Preissner R. (2018). Prediction is a balancing act: Importance of sampling methods to balance sensitivity and specificity of predictive models based on imbalanced chemical data sets. Front. Chem. 6, 362. 10.3389/fchem.2018.00362 30271769 PMC6149243

[B4] Barras D. (2015). BRAF mutation in colorectal cancer: An update. Biomark. Cancer 7 (1), BIC.S25248. 10.4137/BIC.S25248 PMC4562608 26396549

[B5] Batool M. Ahmad B. Choi S. (2019). A structure-based drug discovery paradigm. Int. J. Mol. Sci. 20 (11), 2783. 10.3390/IJMS20112783 PMC6601033 31174387

[B6] Benedetti M. S. Whomsley R. Poggesi I. Cawello W. Mathy F. X. Delporte M. L. (2009). Drug metabolism and pharmacokinetics. Drug Metab. Rev. 41 (3), 344–390. 10.1080/1083745090289129510.1080/10837450902891295 19601718

[B7] Bouback T. A. Pokhrel S. Albeshri A. Aljohani A. M. Samad A. Alam R. (2021). Pharmacophore-based virtual screening, quantum mechanics calculations, and molecular dynamics simulation approaches identified potential natural antiviral drug candidates against MERS-CoV S1-NTD. Molecules 26 (16), 4961. 10.3390/MOLECULES26164961 34443556 PMC8401589

[B8] Bowers K. J. Chow E. Xu H. Dror R. O. Eastwood M. P. Gregersen B. A. (2006). Molecular dynamics---Scalable algorithms for molecular dynamics simulations on commodity clusters. Proc. 2006 ACM/IEEE Conf. Supercomput. 06, 84. 10.1145/1188455.1188544

[B9] Cardarella S. Ogino A. Nishino M. Butaney M. Shen J. Lydon C. (2013). Clinical, pathologic, and biologic features associated with *BRAF* mutations in non–small cell lung cancer. Clin. Cancer Res. 19 (16), 4532–4540. 10.1158/1078-0432.CCR-13-0657 23833300 PMC3762878

[B10] Cope N. Candelora C. Wong K. Kumar S. Nan H. Grasso M. (2018). Mechanism of BRAF activation through biochemical characterization of the recombinant full-length protein. ChemBioChem 19 (18), 1988–1997. 10.1002/CBIC.201800359 29992710 PMC6504641

[B11] Daina A. Michielin O. Zoete V. (2014). Ilogp: A simple, robust, and efficient description of n-octanol/water partition coefficient for drug design using the GB/SA approach. J. Chem. Inf. Model. 54 (12), 3284–3301. 10.1021/ci500467k 25382374

[B12] Daina A. Michielin O. Zoete V. (2017). SwissADME: A free web tool to evaluate pharmacokinetics, drug-likeness and medicinal chemistry friendliness of small molecules. Sci. Rep. 7, 42717. 10.1038/srep42717 28256516 PMC5335600

[B13] Dallakyan S. Olson A. J. (2015). Small-molecule library screening by docking with PyRx. Methods Mol. Biol. 1263, 243–250. 10.1007/978-1-4939-2269-7_19 25618350

[B14] Eroglu Z. Ribas A. (2016). Combination therapy with BRAF and MEK inhibitors for melanoma: Latest evidence and place in therapy. Ther. Adv. Med. Oncol. 8 (1), 48–56. 10.1177/1758834015616934 26753005 PMC4699264

[B15] Fanelli G. N. Dal Pozzo C. A. Depetris I. Schirripa M. Brignola S. Biason P. (2020). The heterogeneous clinical and pathological landscapes of metastatic Braf-mutated colorectal cancer. Cancer Cell Int. 20 (11), 30–12. 10.1186/S12935-020-1117-2 32015690 PMC6990491

[B16] Gaulton A. Hersey A. Nowotka M. L. Patricia Bento A. Chambers J. Mendez D. (2017). The ChEMBL database in 2017. Nucleic Acids Res. 45 (D1), D945–D954. 10.1093/NAR/GKW1074 27899562 PMC5210557

[B17] Geel R. M. J. M. van Iersel L. B. J. V. (2022). Combined targeted therapy for BRAF mutant metastatic colorectal cancer: Are we there yet? Dig. Med. Res. 5 (0), 5. 10.21037/DMR-22-15

[B18] Genheden S. Ryde U. (2015). The MM/PBSA and MM/GBSA methods to estimate ligand-binding affinities. Expert Opin. Drug Discov. 10 (5), 449–461. 10.1517/17460441.2015.1032936 25835573 PMC4487606

[B19] Grassi E. Corbelli J. Papiani G. Barbera M. A. Gazzaneo F. Tamberi S. (2021). Current therapeutic strategies in BRAF-mutant metastatic colorectal cancer. Front. Oncol. 11, 601722. 10.3389/fonc.2021.601722 34249672 PMC8262685

[B20] Gray M. E. Johnson Z. R. Modak D. Tamilselvan E. Tyska M. J. Sotomayor M. (2021). A crowdsourcing open platform for literature curation in UniProt. PLoS Biol. 19 (12), e3001464. 10.1371/JOURNAL.PBIO.3001464 34871295 PMC8675915

[B21] Guo Y. Pan W. Liu S. Shen Z. Xu Y. Hu L. (2020). ERK/MAPK signalling pathway and tumorigenesis. Exp. Ther. Med. 19 (3), 1997–2007. 10.3892/ETM.2020.8454 32104259 PMC7027163

[B22] Holderfield M. Deuker M. M. McCormick F. McMahon M. (2014). Targeting RAF kinases for cancer therapy: BRAF mutated melanoma and beyond. Nat. Rev. Cancer 14 (7), 455–467. 10.1038/NRC3760 24957944 PMC4250230

[B23] Hussain M. R. M. Baig M. Mohamoud H. S. A. Ulhaq Z. Hoessli D. C. Khogeer G. S. (2015). BRAF gene: From human cancers to developmental syndromes. Saudi J. Biol. Sci. 22 (4), 359–373. 10.1016/J.SJBS.2014.10.002 26150740 PMC4486731

[B24] Hwang S. B. Lee C. J. Lee S. Ma S. Kang Y. M. Cho K. H. (2020). Pmff: Development of a physics-based molecular force field for protein simulation and ligand docking. J. Phys. Chem. B 124 (6), 974–989. 10.1021/ACS.JPCB.9B10339/ASSET/IMAGES/LARGE/JP9B10339_0001 31939671 PMC7217328

[B25] Irwin J. J. Tang K. G. Young J. Dandarchuluun C. Wong B. R. Khurelbaatar M. (2020). ZINC20 – a free ultra large-scale chemical database for ligand discovery. J. Chem. Inf. Model. 60 (12), 6065–6073. 10.1021/ACS.JCIM.0C00675 33118813 PMC8284596

[B26] Jendele L. Krivak R. Skoda P. Novotny M. Hoksza D. (2019). PrankWeb: A web server for ligand binding site prediction and visualization. Nucleic Acids Res. 47 (W1), W345–W349. 10.1093/NAR/GKZ424 31114880 PMC6602436

[B27] Jin Z. Wang Y. Yu X. F. Tan Q. Q. Liang S. S. Li T. (2020). Structure-based virtual screening of influenza virus RNA polymerase inhibitors from natural compounds: Molecular dynamics simulation and MM-GBSA calculation. Comput. Biol. Chem. 85, 107241. 10.1016/J.COMPBIOLCHEM.2020.107241 32120300

[B28] Kim A. Cohen M. S. (2016). The discovery of vemurafenib for the treatment of BRAF-mutated metastatic melanoma. Expert Opin. Drug Discov. 11 (9), 907–916. 10.1080/17460441.2016.1201057 27327499 PMC5443413

[B29] Kim S. Chen J. Cheng T. Gindulyte A. He J. He S. (2021). PubChem in 2021: New data content and improved web interfaces. Nucleic Acids Res. 49 (D1), D1388–D1395. 10.1093/NAR/GKAA971 33151290 PMC7778930

[B30] Koes D. R. Camacho C. J. (2012). ZINCPharmer: Pharmacophore search of the ZINC database. Nucleic Acids Res. 40 (W1), W409–W414. 10.1093/nar/gks378 22553363 PMC3394271

[B31] Lasota J. Kowalik A. Wasag B. Wang Z. F. Felisiak-Golabek A. Coates T. (2014). Detection of the BRAF V600E mutation in colon carcinoma – critical evaluation of the imunohistochemical approach. Am. J. Surg. Pathol. 38 (9), 1235–1241. 10.1097/PAS.0000000000000229 24832158 PMC4134735

[B32] Leonetti A. Facchinetti F. Rossi G. Minari R. Conti A. Friboulet L. (2018). BRAF in non-small cell lung cancer (NSCLC): Pickaxing another brick in the wall. Cancer Treat. Rev. 66, 82–94. 10.1016/J.CTRV.2018.04.006 29729495

[B33] Luu L.-J. Price T. J. (2019). BRAF mutation and its importance in colorectal cancer. Adv. Mol. Underst. Colorectal Cancer 7, 9–12. 10.5772/INTECHOPEN.82571

[B34] McCubrey J. A. Steelman L. S. Chappell W. H. Abrams S. L. Wong E. W. T. Chang F. (2007). Roles of the raf/mek/erk pathway in cell growth, malignant transformation and drug resistance. Biochimica Biophysica Acta - Mol. Cell Res. 1773 (8), 1263–1284. 10.1016/J.BBAMCR.2006.10.001 PMC2696318 17126425

[B35] Menzies A. M. Haydu L. E. Visintin L. Carlino M. S. Howle J. R. Thompson J. F. (2012). Distinguishing clinicopathologic features of patients with V600E and V600K BRAF-mutant metastatic melanoma. Clin. Cancer Res. 18 (12), 3242–3249. 10.1158/1078-0432.CCR-12-0052 22535154

[B36] Merz V. Gaule M. Zecchetto C. Cavaliere A. Casalino S. Pesoni C. (2021). Targeting KRAS: The elephant in the room of epithelial cancers. Front. Oncol. 11, 638360. 10.3389/fonc.2021.638360 33777798 PMC7991835

[B37] Morkel M. Riemer P. Bläker H. Sers C. (2015). Similar but different: Distinct roles for KRAS and BRAF oncogenes in colorectal cancer development and therapy resistance. Oncotarget 6 (25), 20785–20800. 10.18632/ONCOTARGET.4750 26299805 PMC4673229

[B38] Mysinger M. M. Carchia M. Irwin J. J. Shoichet B. K. (2012). Directory of useful decoys, enhanced (DUD-E): Better ligands and decoys for better benchmarking. J. Med. Chem. 55 (14), 6582–6594. 10.1021/jm300687e 22716043 PMC3405771

[B39] Nguyen-Ngoc T. Bouchaab H. Adjei A. A. Peters S. (2015). BRAF alterations as therapeutic targets in non–small-cell lung cancer. J. Thorac. Oncol. 10 (10), 1396–1403. 10.1097/JTO.0000000000000644 26301799

[B40] Nishiguchi G. A. Rico A. Tanner H. Aversa R. J. Taft B. R. Subramanian S. (2017). Design and discovery of N-(2-Methyl-5’-morpholino-6’-((tetrahydro-2H-pyran-4-yl)oxy)-[3, 3’-bipyridin]-5-yl)-3-(trifluoromethyl)benzamide (RAF709): A potent, selective, and efficacious RAF inhibitor targeting RAS mutant cancers. J. Med. Chem. 60 (12), 4869–4881. 10.1021/ACS.JMEDCHEM.6B01862 28557458

[B41] Opo F. A. D. M. Rahman M. M. Ahammad F. Ahmed I. Bhuiyan M. A. Asiri A. M. (2021). Structure based pharmacophore modeling, virtual screening, molecular docking and ADMET approaches for identification of natural anti-cancer agents targeting XIAP protein. Sci. Rep. 11 (1), 4049–4117. 10.1038/s41598-021-83626-x 33603068 PMC7892887

[B42] Ormö M. Cubitt A. B. Kallio K. Gross L. A. Tsien R. Y. Remington S. J. (1996). Crystal structure of the Aequorea victoria green fluorescent protein. Sci. (New York, N.Y.) 273 (5280), 1392–1395. 10.1126/SCIENCE.273.5280.1392 8703075

[B43] Patel H. Yacoub N. Mishra R. White A. Yuan L. Alanazi S. (2020). Current advances in the treatment of BRAF-mutant melanoma. Cancers 12 (2), 482. 10.3390/CANCERS12020482 PMC7072236 32092958

[B44] Proietti I. Skroza N. Michelini S. Mambrin A. Balduzzi V. Bernardini N. (2020). BRAF inhibitors: Molecular targeting and immunomodulatory actions. Cancers 12 (7), 1823–1913. 10.3390/CANCERS12071823 PMC7408709 32645969

[B45] Rella M. Rushworth C. A. Guy J. L. Turner A. J. Langer T. Jackson R. M. (2006). Structure-based pharmacophore design and virtual screening for novel Angiotensin Converting Enzyme 2 inhibitors. J. Chem. Inf. Model. 46 (2), 708–716. 10.1021/ci0503614 16563001

[B46] Richards A. Garg V. (2010). Genetics of congenital heart disease. Curr. Cardiol. Rev. 6 (2), 91–97. 10.2174/157340310791162703 21532774 PMC2892081

[B47] Roviello G. D’Angelo A. Petrioli R. Roviello F. Cianchi F. Nobili S. (2020). Encorafenib, binimetinib, and cetuximab in BRAF V600e-mutated colorectal cancer. Transl. Oncol. 13 (9), 100795. 10.1016/J.TRANON.2020.100795 32470910 PMC7260582

[B48] Rowe L. R. Bentz B. G. Bentz J. S. (2007). Detection of BRAF V600E activating mutation in papillary thyroid carcinoma using PCR with allele‐specific fluorescent probe melting curve analysis. J. Clin. Pathology 60 (11), 1211–1215. 10.1136/JCP.2006.040105 PMC2095462 17298986

[B49] Salmaso V. Moro S. (2018). Bridging molecular docking to molecular dynamics in exploring ligand-protein recognition process: An overview. Front. Pharmacol. 9, 923. 10.3389/fphar.2018.00923 30186166 PMC6113859

[B50] Shivakumar D. Williams J. Wu Y. Damm W. Shelley J. Sherman W. (2010). Prediction of absolute solvation free energies using molecular dynamics free energy perturbation and the opls force field. J. Chem. Theory Comput. 6 (5), 1509–1519. 10.1021/ct900587b 26615687

[B51] Stillhart C. Vučićević K. Augustijns P. Basit A. W. Batchelor H. Flanagan T. R. (2020). Impact of gastrointestinal physiology on drug absorption in special populations––An UNGAP review. Eur. J. Pharm. Sci. 147, 105280. 10.1016/J.EJPS.2020.105280 32109493

[B52] Tanda E. T. Vanni I. Boutros A. Andreotti V. Bruno W. Ghiorzo P. (2020). Current state of target treatment in BRAF mutated melanoma. Front. Mol. Biosci. 7, 154. 10.3389/fmolb.2020.00154 32760738 PMC7371970

[B53] Tian W. Chen C. Lei X. Zhao J. Liang J. (2018). CASTp 3.0: Computed atlas of surface topography of proteins. Nucleic Acids Res. 46 (W1), W363–W367. 10.1093/nar/gky473 29860391 PMC6031066

[B54] Tran T. V. Dang K. X. Pham Q. H. Nguyen U. D. Trinh N. T. T. Hoang L. Van (2020). Evaluation of the expression levels of BRAF V600E mRNA in primary tumors of thyroid cancer using an ultrasensitive mutation assay. BMC Cancer 20 (1), 368–369. 10.1186/s12885-020-06862-w 32357861 PMC7195771

[B55] Tufano R. P. Teixeira G. V. Bishop J. Carson K. A. Xing M. (2012). BRAF mutation in papillary thyroid cancer and its value in tailoring initial treatment: A systematic review and meta-analysis. Med. (United States) 91 (5), 274–286. 10.1097/MD.0B013E31826A9C71 22932786

[B56] Turashvili G. Grisham R. N. Chiang S. DeLair D. F. Park K. J. Soslow R. A. (2018). *BRAF* ^<i>V</i><i>600E</i>^mutations and immunohistochemical expression of VE1 protein in low-grade serous neoplasms of the ovary. Histopathology 73 (3), 438–443. 10.1111/HIS.13651 29770477 PMC6105553

[B57] Valasani K. R. Vangavaragu J. R. Day V. W. Yan S. S. (2014). Structure based design, synthesis, pharmacophore modeling, virtual screening, and molecular docking studies for identification of novel cyclophilin D inhibitors. J. Chem. Inf. Model. 54 (3), 902–912. 10.1021/ci5000196 24555519 PMC3985759

[B58] Varma A. K. Patil R. Das S. Stanley A. Yadav L. Sudhakar A. (2010). Optimized hydrophobic interactions and hydrogen bonding at the target-ligand interface leads the pathways of drug-designing. PLoS ONE 5 (8), e12029. 10.1371/JOURNAL.PONE.0012029 20808434 PMC2922327

[B59] Wang L. Lu Q. Jiang K. Hong R. Wang S. Xu F. (2022). BRAF V600E mutation in triple-negative breast cancer: A case report and literature review. Oncol. Res. Treat. 45 (1–2), 54–61. 10.1159/000520453 34818649 PMC8985016

[B60] Watanabe R. Ohashi R. Esaki T. Kawashima H. Natsume-Kitatani Y. Nagao C. (2019). Development of an *in silico* prediction system of human renal excretion and clearance from chemical structure information incorporating fraction unbound in plasma as a descriptor. Sci. Rep. 9 (1), 18782. 10.1038/S41598-019-55325-1 31827176 PMC6906481

[B61] Wolber G. Langer T. (2005). LigandScout: 3-D pharmacophores derived from protein-bound ligands and their use as virtual screening filters. J. Chem. Inf. Model. 45 (1), 160–169. 10.1021/ci049885e 15667141

[B62] Yan N. Guo S. Zhang H. Zhang Z. Shen S. Li X. (2022). BRAF-mutated non-small cell lung cancer: Current treatment status and future perspective. Front. Oncol. 12, 863043. 10.3389/FONC.2022.863043 35433454 PMC9008712

