# Supplementary material for: Identification of novel natural drug candidates against BRAF mutated carcinoma; An integrative in-silico structure-based pharmacophore modeling and virtual screening process
Source: Front Chem. 2022 Oct 4;10:986376. doi: 10.3389/fchem.2022.986376 (PMC9577413; doi:10.3389/fchem.2022.986376)
Supplement: Supplementary file 1 [file DataSheet1.docx]

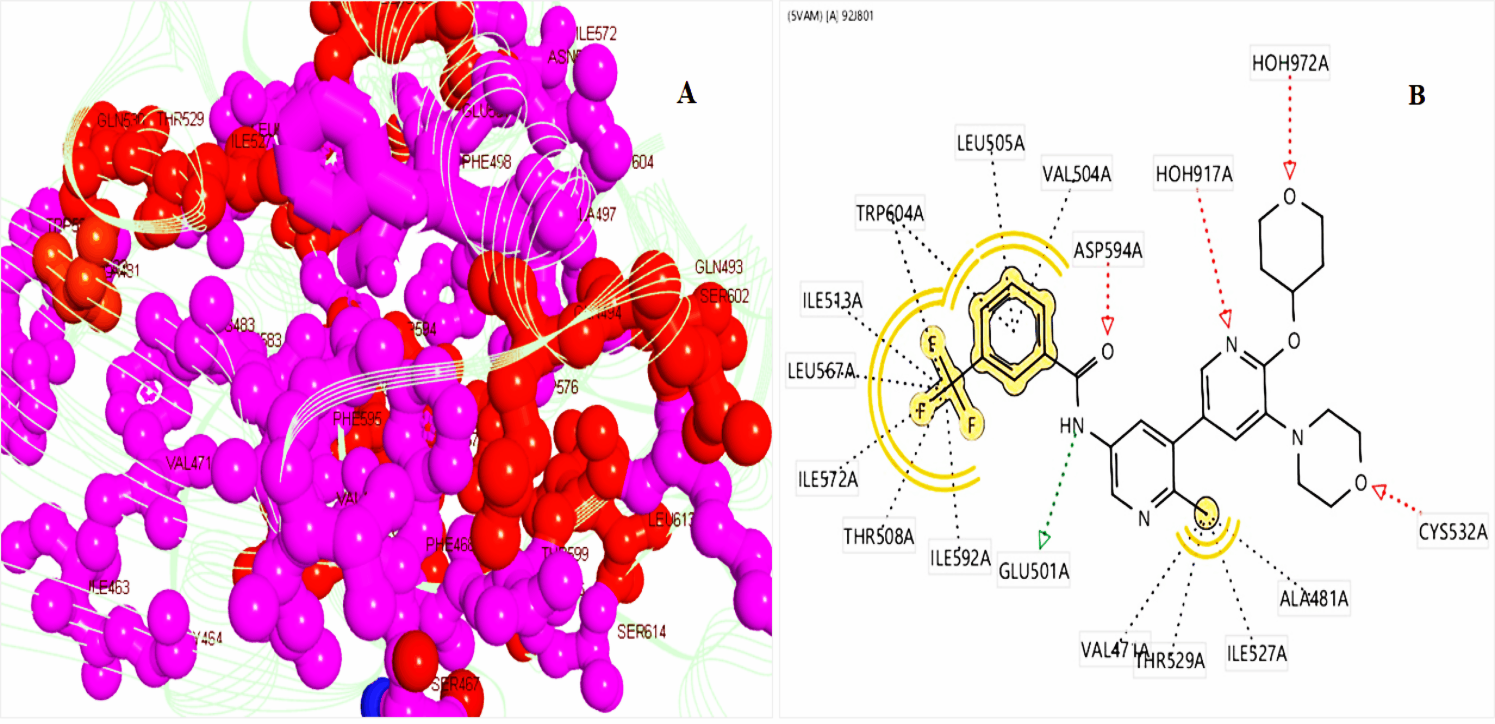


**Figure S1:** The active site (A) identification for the selected protein by the CASTp server and the 2D interaction (B) of the ligand with the protein determined by the Ligand Scout 4.4.8


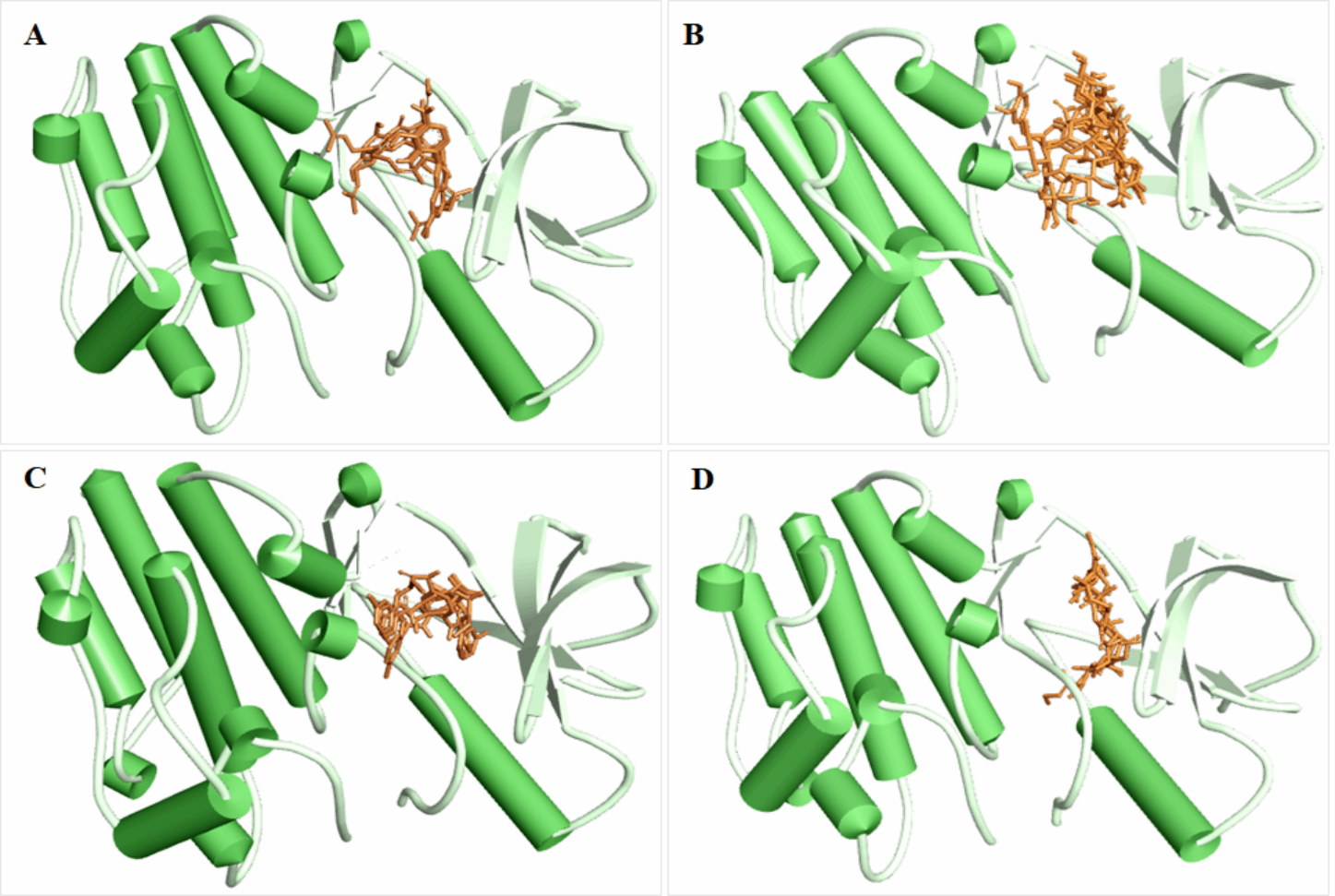


**Figure S2:** Validation of the each selected anatagonist with our target protein (PDB ID: 5VAM)


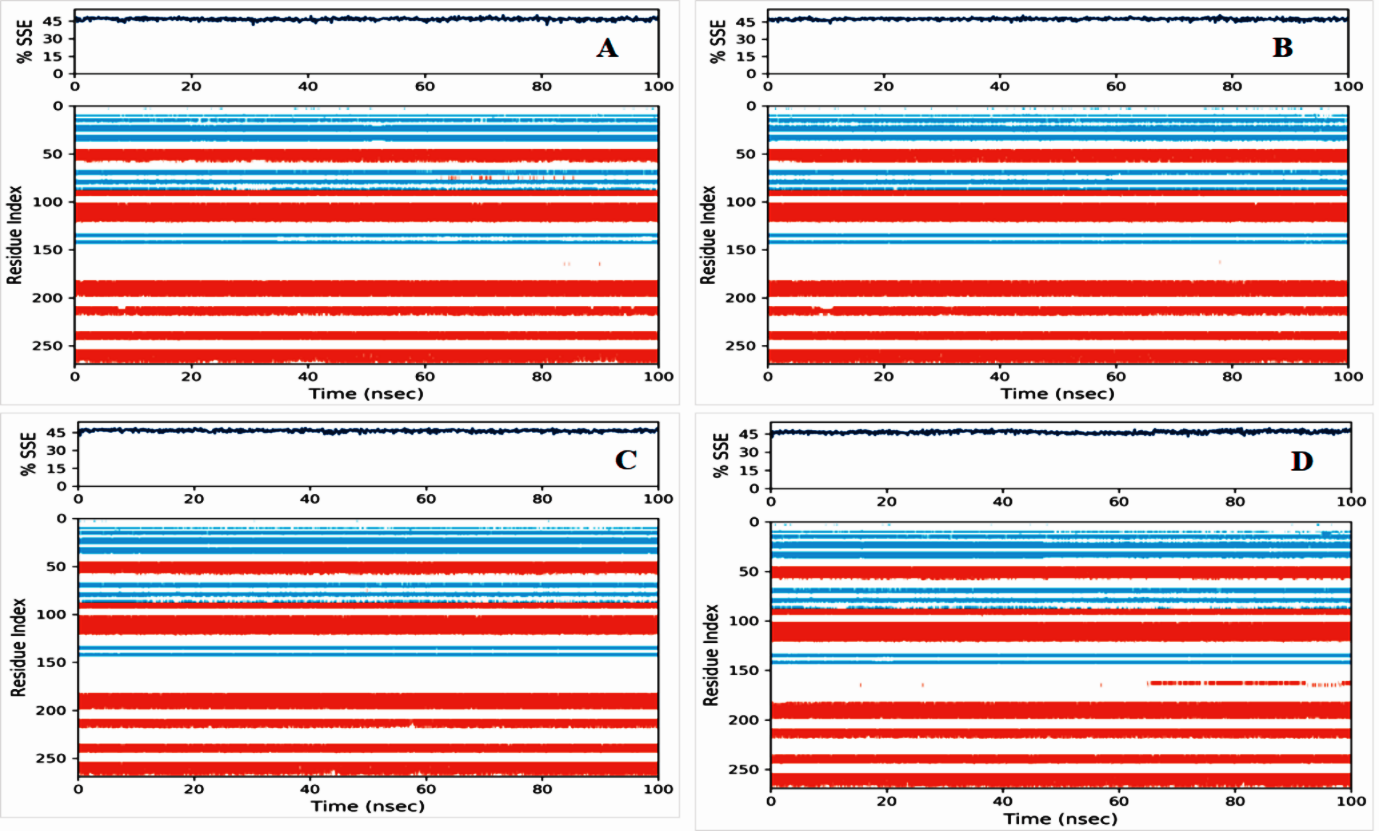


**Figure S3:** The plot tracks every residue's SSE assignment over time whereas the plot above indicated the SSE composition for every trajectory frames across the simulation.


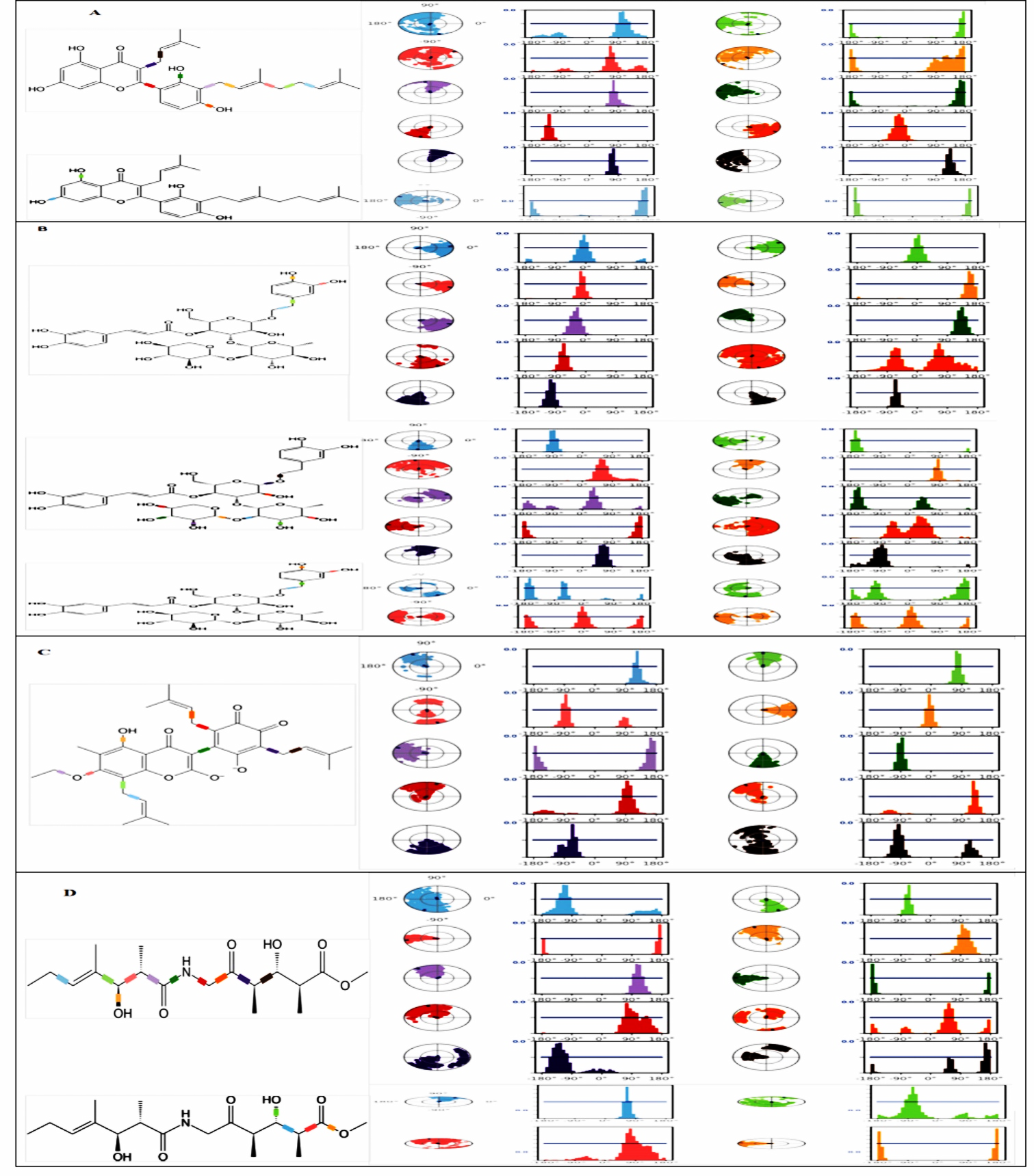


**Figure S4:** Torsional confirmation of the each rotatable bonds of the selected antagonist compound. Schematic 2D diagram showed that the colour coated rotatable bonds of ligands (Left). Radial charts showed how the torsion has changed throughout the time of simulation. Bar plots described the probability density of the toriosn (Right)

**Table S1:** The Pub Chem CID, Molecular weight, and IC_50_ of known antagonists against the BRAF protein are listed here. The docking score has been combined with the binding affinity of each antagonist.

| **PubChem CID** | **Molecular Formula** | **Chemical Structure** | **Molecular Weight** | **IC_50_ (uM)** | **Binding Affinity (kcal/mol)** |
| --- | --- | --- | --- | --- | --- |
| 58087865 | [C_23_H_19_F_2_N_3_O_3_S](https://pubchem.ncbi.nlm.nih.gov/#query=C23H19F2N3O3S) | 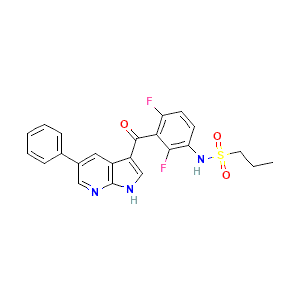 | 455.5 | 0.04 | -8.1 |
| 145971184 | [C_28_H_21_N_3_O_3_](https://pubchem.ncbi.nlm.nih.gov/#query=C28H21N3O3) | 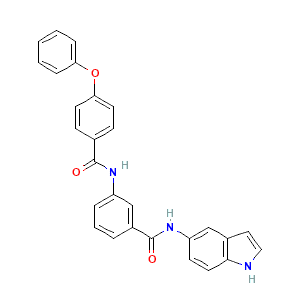 | 447.5 | 5.72 | -8.0 |
| 50922675 | [C_22_H_27_ClFN_7_O_4_S](https://pubchem.ncbi.nlm.nih.gov/#query=C22H27ClFN7O4S) | 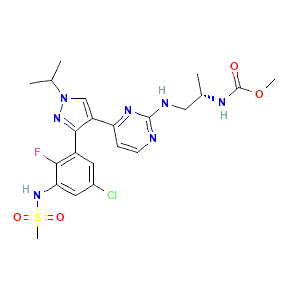 | 540.0 | 0.0003 | -8.1 |
| 46228554 | [C_19_H_14_ClN_3_O_4_S](https://pubchem.ncbi.nlm.nih.gov/#query=C19H14ClN3O4S) | 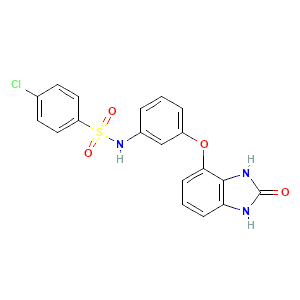 | 415.9 | 2.5 | -7.7 |
| 46228666 | [C_24_H_17_N_3_O_3_](https://pubchem.ncbi.nlm.nih.gov/#query=C24H17N3O3) | 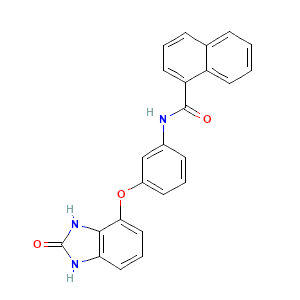 | 395.4 | 0.92 | -8.0 |
| 46228951 | [C_21_H_11_F_6_N_3_O_4_](https://pubchem.ncbi.nlm.nih.gov/#query=C21H11F6N3O4) | 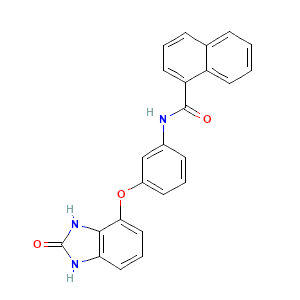 | 483.3 | 0.042 | -8.1 |
| 46228819 | [C_20_H_14_F_3_N_3_O_4_S](https://pubchem.ncbi.nlm.nih.gov/#query=C20H14F3N3O4S) | 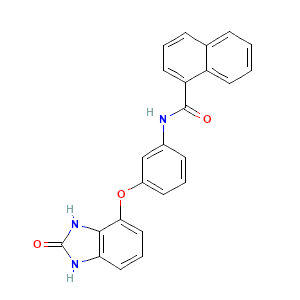 | 449.4 | 15.4 | -7.8 |
| 46228767 | [C_27_H_25_N_5_O_3_](https://pubchem.ncbi.nlm.nih.gov/#query=C27H25N5O3) | 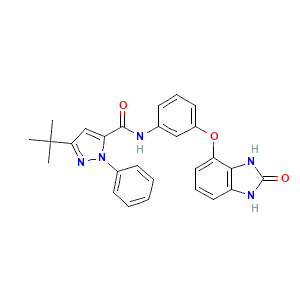 | 467.5 | 0.274 | -7.9 |
| 46228527 | [C_20_H_12_ClF_4_N_3_O_4_S](https://pubchem.ncbi.nlm.nih.gov/#query=C20H12ClF4N3O4S) | 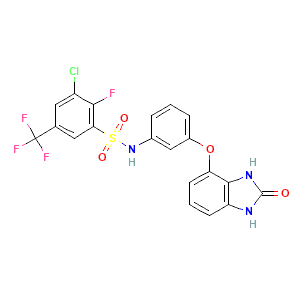 | 501.8 | 0.62 | -8.1 |
| 46228590 | [C_21_H_13_F_4_N_3_O_4_](https://pubchem.ncbi.nlm.nih.gov/#query=C21H13F4N3O4) | 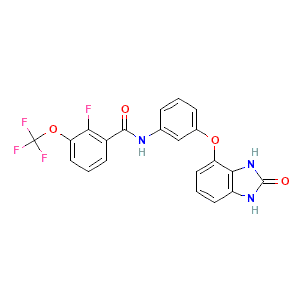 | 447.3 | 0.015 | -8.0 |
| 46228951 | [C_21_H_11_F_6_N_3_O_4_](https://pubchem.ncbi.nlm.nih.gov/#query=C21H11F6N3O4) | 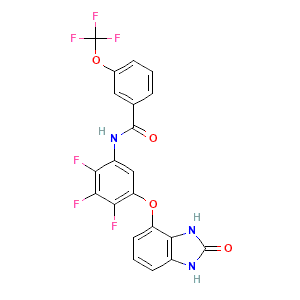 | 483.3 | 0.007 | -7.4 |
| 11167602 | [C_21_H_15_ClF_4_N_4_O_3_](https://pubchem.ncbi.nlm.nih.gov/#query=C21H15ClF4N4O3) | 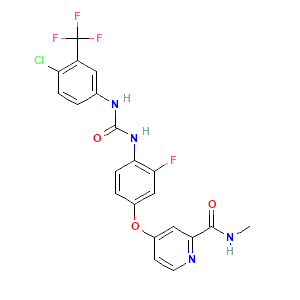 | 482.8 | 0.0015 | -7.2 |
| 56931136 | [C_24_H_19_ClN_4_O_4_](https://pubchem.ncbi.nlm.nih.gov/#query=C24H19ClN4O4) | 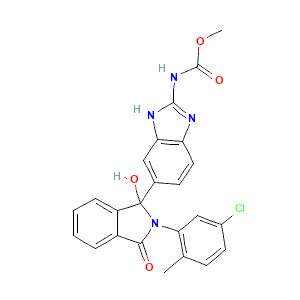 | 462.9 | 2 | -7.0 |
| 44462760 | [C_23_H_20_F_3_N_5_O_2_S_2_](https://pubchem.ncbi.nlm.nih.gov/#query=C23H20F3N5O2S2) | 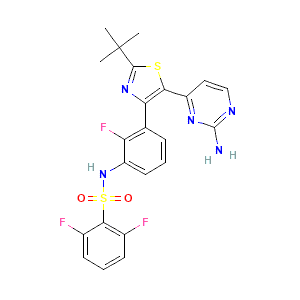 | 519.6 | 0.0007 | -7.3 |
| 90408826 | [C_28_H_29_F_3_N_4_O_4_](https://pubchem.ncbi.nlm.nih.gov/#query=C28H29F3N4O4) | 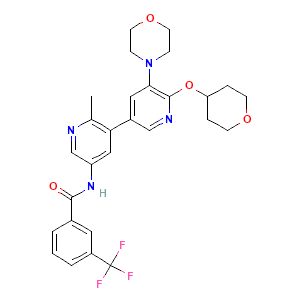 | 542.5 | 0.0004 | -8.0 |
| 42611257 | [C_23_H_18_ClF_2_N_3_O_3_S](https://pubchem.ncbi.nlm.nih.gov/#query=C23H18ClF2N3O3S) | 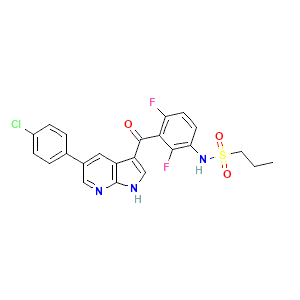 | 489.9 | 0.0032 | -7.5 |
| 25071591 | [C_26_H_25_ClF_3_N_5_O_5_](https://pubchem.ncbi.nlm.nih.gov/#query=C26H25ClF3N5O5) | 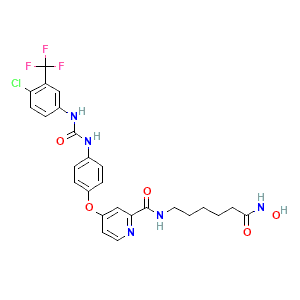 | 580.0 | 1.0 | -7.6 |
| 145966312 | [C_18_H_13_ClN_2_O](https://pubchem.ncbi.nlm.nih.gov/#query=C18H13ClN2O) | 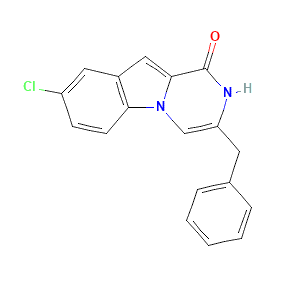 | 308.8 | 1.0 | -7.1 |
| 145988888 | [C_24_H_20_BrN_3_O_2_S](https://pubchem.ncbi.nlm.nih.gov/#query=C24H20BrN3O2S) | 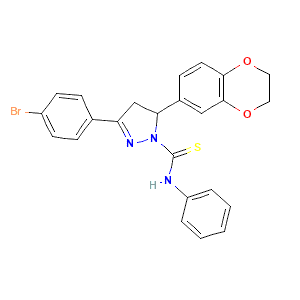 | 494.4 | 0.05 | -7.7 |
| 145990447 | [C_22_H_19_N_3_O_2_S_2_](https://pubchem.ncbi.nlm.nih.gov/#query=C22H19N3O2S2) | 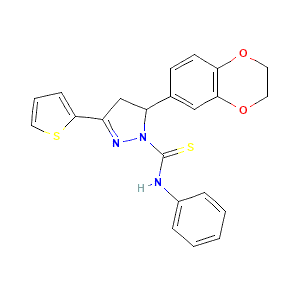 | 421.5 | 5.21 | -7.1 |
| 145969111 | [C_21_H_15_BrN_4_O_2_](https://pubchem.ncbi.nlm.nih.gov/#query=C21H15BrN4O2) | 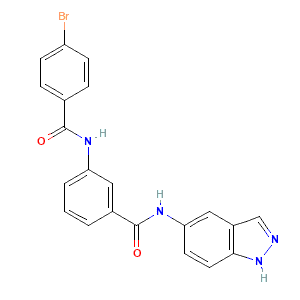 | 435.3 | 2.78 | -8.1 |
| 145981655 | [C_17_H_12_ClN_3_OS](https://pubchem.ncbi.nlm.nih.gov/#query=C17H12ClN3OS) | 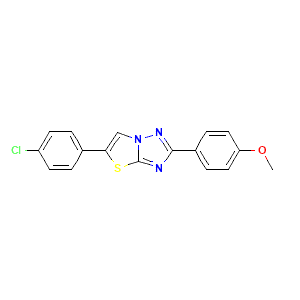 | 341.8 | 1.5 | -6.4 |
| 145967582 | [C_20_H_14_ClN_5_O_2_](https://pubchem.ncbi.nlm.nih.gov/#query=C20H14ClN5O2) | 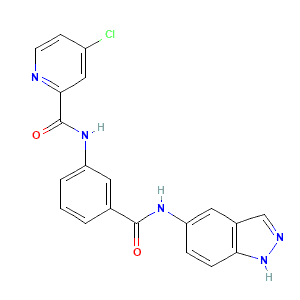 | 391.8 | 1.14 | -7.5 |
| 52900120 | [C_14_H_14_N_4_O](https://pubchem.ncbi.nlm.nih.gov/#query=C14H14N4O) | 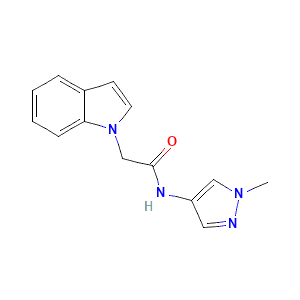 | 254.29 | 4.66 | -6.8 |
| 141484716 | [C_20_H_18_N_6_O_2_](https://pubchem.ncbi.nlm.nih.gov/#query=C20H18N6O2) | 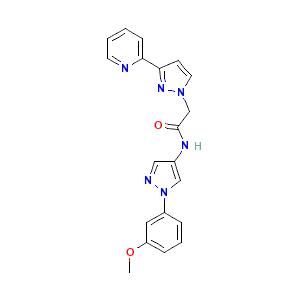 | 374.4 | 0.26 | -7.3 |

**Table S2:** The list of active site determined from the online database CASTp server

| **Pocket ID** | **Chain** | **Sequence ID** | **Amino Acid** | **Atom** |
| --- | --- | --- | --- | --- |
| 1 | A | 463 | ILE | CG2 |
| 1 | A | 464 | GLY | CA |
| 1 | A | 467 | SER | O |
| 1 | A | 468 | PHE | O |
| 1 | A | 471 | VAL | CB |
| 1 | A | 481 | ALA | C |
| 1 | A | 483 | LYS | N |
| 1 | A | 485 | LEU | CD2 |
| 1 | A | 486 | ASN | CB |
| 1 | A | 487 | VAL | CG2 |
| 1 | A | 493 | GLN | C |
| 1 | A | 494 | GLN | NE2 |
| 1 | A | 497 | ALA | CA |
| 1 | A | 498 | PHE | CA |
| 1 | A | 500 | ASN | CB |
| 1 | A | 501 | GLU | O |
| 1 | A | 504 | VAL | CG1 |
| 1 | A | 505 | LEU | CG |
| 1 | A | 508 | THR | CG2 |
| 1 | A | 513 | ILE | CG2 |
| 1 | A | 514 | LEU | CB |
| 1 | A | 527 | ILE | O |
| 1 | A | 529 | THR | CG2 |
| 1 | A | 530 | GLN | O |
| 1 | A | 531 | TRP | CA |
| 1 | A | 532 | CYS | SG |
| 1 | A | 567 | LEU | CD1 |
| 1 | A | 572 | ILE | CG2 |
| 1 | A | 574 | HIS | NE2 |
| 1 | A | 576 | ASP | OD2 |
| 1 | A | 578 | LYS | NZ |
| 1 | A | 581 | ASN | ND2 |
| 1 | A | 583 | PHE | CB |
| 1 | A | 592 | ILE | CG2 |
| 1 | A | 593 | GLY | CA |
| 1 | A | 594 | ASP | OD2 |
| 1 | A | 595 | PHE | CA |
| 1 | A | 596 | GLY | O |
| 1 | A | 597 | LEU | CA |
| 1 | A | 598 | ALA | CB |
| 1 | A | 599 | THR | CA |
| 1 | A | 600 | VAL | CG2 |
| 1 | A | 601 | LYS | CA |
| 1 | A | 602 | SER | CA |
| 1 | A | 603 | ARG | N |
| 1 | A | 604 | TRP | CH2 |
| 1 | A | 613 | LEU | CA |
| 1 | A | 614 | SER | OG |
| 1 | A | 616 | SER | OG |
